# Enhancing the production of cephalosporin C through modulating the autophagic process of *Acremonium chrysogenum*

**DOI:** 10.1186/s12934-018-1021-9

**Published:** 2018-11-13

**Authors:** Honghua Li, Pengjie Hu, Ying Wang, Yuanyuan Pan, Gang Liu

**Affiliations:** 1 State Key Laboratory of Mycology, Institute of Microbiology, Chinese Academy of Sciences, Beijing, 100101 China; 2 University of Chinese Academy of Sciences, Beijing, 100049 China

**Keywords:** *Acatg8*, *Acremonium chrysogenum*, Autophagy, Cephalosporin C, Conidial germination

## Abstract

**Background:**

Autophagy is used for degradation of cellular components and nutrient recycling. Atg8 is one of the core proteins in autophagy and used as a marker for autophagic detection. However, the autophagy of filamentous fungi is poorly understood compared with that of *Saccharomyces cerevisiae*. Our previous study revealed that disruption of the autophagy related gene *Acatg1* significantly enhanced cephalosporin C yield through reducing degradation of cephalosporin biosynthetic proteins in *Acremonium chrysogenum*, suggesting that modulation of autophagic process is one promising way to increase antibiotic production in *A. chrysogenum*.

**Results:**

In this study, a *S. cerevisiae ATG8* homologue gene *Acatg8* was identified from *A. chrysogenum*. *Acatg8* could complement the *ATG8* mutation in *S. cerevisiae*, indicating that *Acatg8* is a functional homologue of *ATG8*. Microscope observation demonstrated the fluorescently labeled AcAtg8 was localized in the cytoplasm and autophagosome of *A. chrysogenum*, and the expression of *Acatg8* was induced by nutrient starvation. Gene disruption and genetic complementation revealed that *Acatg8* is essential for autophagosome formation. Disruption of *Acatg8* significantly reduced fungal conidiation and delayed conidial germination. Localization of GFP-AcAtg8 implied that autophagy is involved in the early phase of conidial germination. Similar to *Acatg1*, disruption of *Acatg8* remarkably enhanced cephalosporin C yield. The cephalosporin C biosynthetic enzymes (isopenicillin N synthase PcbC and isopenicillin N epimerase CefD2) and peroxisomes were accumulated in the *Acatg8* disruption mutant (∆Acatg8), which might be the main reasons for the enhancement of cephalosporin C production. However, the biomass of ΔAcatg8 decreased drastically at the late stage of fermentation, suggesting that autophagy is critical for *A. chrysogenum* cell survival under nutrition deprived condition. Disruption of *Acatg8* also resulted in accumulation of mitochondria, which might produce more reactive oxygen species (ROS) which promotes fungal death. However, the premature death is unfavorable for cephalosporin C production. To solve this problem, a plasmid containing *Acatg8* under control of the xylose/xylan-inducible promoter was introduced into ∆Acatg8. Conidiation and growth of the recombinant strain restored to the wild-type level in the medium supplemented with xylose, while the cephalosporin C production maintained at a high level even prolonged fermentation.

**Conclusions:**

Our results demonstrated inducible expression of *Acatg8* and disruption of *Acatg8* remarkably increased cephalosporin C production. This study provides a promising approach for yield improvement of cephalosporin C in *A. chrysogenum.*

**Electronic supplementary material:**

The online version of this article (10.1186/s12934-018-1021-9) contains supplementary material, which is available to authorized users.

## Background

Macroautophagy (hereafter autophagy) is generally used for degradation of cellular components and nutrient recycling in eukaryotes [1, 2]. The degradation of cytoplasm components (such as cytosol, macromolecular complexes, and organelles) takes place in vacuole/lysosome of eukaryotes. Autophagy normally occurs at a low constitutive level, but it is up-regulated under nutrient starvation condition. Autophagy begins with the formation of a sequestering membrane termed phagophore, and the phagophore expands and forms a double-membrane-bound vesicle known as autophagosome. The autophagosomes enter the vacuoles through membrane fusing and form autophagic bodies in which autophagic cargoes are degraded. As one of the core proteins, the ubiquitin-like protein Atg8 is essential for autophagosome formation and highly conserved in eukaryotes. Besides, Atg8 is also used as a biological marker for autophagic detection [3, 4].

In mammals, autophagy is involved in carcinogenesis, neurodegenerative diseases and developmental processes [5, 6]. Autophagy has been extensively studied in *Saccharomyces cerevisiae*, where more than 40 genes are involved in this process. In *S. cerevisiae*, autophagy deficient mutants not only show a reduction of cell viability under starvation condition, but also display defect in sporulation [7]. Recently, autophagy of filamentous fungi has received more attention since it is involved in fungal survival, reproduction and pathogenicity [8]. In *Magnaporthe grisea*, deletion of the autophagy related genes results in reduction of conidiation and non-pathogenic phenotype [4]. In *Fusarium graminearum*, autophagy is important for lipid turnover, deoxynivalenol production and infection in plant [9]. In the cucumber anthracnose fungus *Colletotrichum orbiculare*, autophagy is required for host invasion [10]. In the well-established aging model *Podospora anserine*, autophagy is related with aging and life span as a longevity-assurance mechanism [11]. In *Aspergillus oryzae*, autophagy is required for the formation of aerial hyphae and conidia [12], and deficiency of autophagy enhances the production of bovine chymosin [13]. In *Sordaria macrospora*, autophagy is used to sustain high energy levels for mycelia growth and morphological differentiation [14]. In *Penicillium chrysogenum*, the *atg1* deletion mutant showed onefold increase in penicillin production [15]. Thus, autophagy is extensively related with morphological differentiation and secondary metabolite productions in filamentous fungi.

*Acremonium chrysogenum* is well known for producing the pharmaceutically relevant β-lactam antibiotic cephalosporin C (CPC). The cephalosporin biosynthetic genes of *A. chrysogenum* are localized in two separated clusters [16]. The CPC biosynthetic pathway has been well studied and at least 6 biosynthetic genes (*pcbAB, pcbC, cefD1, cefD2, cefEF* and *cefG*) are essential for the CPC biosynthesis [17, 18]. Like most of secondary metabolites, cephalosporin C is produced at the anaphase of exponential growth and stationary phase [19, 20]. During this time, the nutrients are depleted and autophagy is induced. It is speculated that the CPC biosynthetic proteins including PcbAB, PcbC, CefD1 and CefD2 could be degraded through autophagic process. In fact, disruption of an autophagy-related serine/threonine kinase gene *Acatg1* significantly enhanced CPC yield through retaining PcbC and increasing the transcriptional levels of related genes [21]. *Acatg1* is essential for the formation of autophagosome under starvation in *A. chrysogenum*. *Acatg11* is involved in the selective autophagy pathway as a basic scaffold for phagosome assembly. However, deficiency of *Acatg11* did not increase CPC production [22]. The relationship between autophagy and protein degradation should be complicated. How exactly autophagy participates in these fungal processes remains unknown.

In the present study, a *S. cerevisiae ATG8* homologue gene *Acatg8* was identified from *A. chrysogenum*. Gene disruption and genetic complementation revealed that *Acatg8* is essential for autophagosome formation and autophagic process. Disruption of *Acatg8* significantly reduced conidiation and fungal viability especially at the late stage of fermentation, but remarkably enhanced cephalosporin C yield. Through induced expression of *Acatg8*, the autophagic process was modulated and cephalosporin C yield was remarkably increased in the case of maintaining fungal viability. This study provides a promising approach for increasing antibiotic yield through modulating autophagic process in *A. chrysogenum.*

## Materials and methods

### Strains, media, and growth conditions

Strains and plasmids used in this study were listed in Additional file 1: Table S1. For growth and conidiation of *A. chrysogenum*, TSA and LPE media were used respectively as described previously [22]. The modified MDFA medium was used for CPC production as described previously [23]. Czapek medium (per liter: 30.0 g sucrose, 3.0 g NaNO_3_, 0.5 g MgSO_4_·7H_2_O, 0.0125 g FeSO_4_·7H_2_O, 0.5 g KCl, 1.3 g K_2_HPO_4_·3H_2_O, 15.0 g agar), Czapek–N medium (Czapek medium without nitrogen source), Czapek–C medium (Czapek medium without carbon source) and WA medium (water with 1% agar) were used to detect the viability of *A. chrysogenum* and its derivatives. For *Agrobacterium tumefaciens*-mediated transformation (ATMT), minimal medium, co-cultivation medium and induction medium were used as described previously [23]. YPD medium (per litter: yeast extract 10.0 g, tryptone 20.0 g, glucose 20.0 g, agar 20.0 g) was used for the growth of *S. cerevisiae* BY4742 (the wild-type strain) and its derivatives. Nitrogen-starved medium (SG-N) (per litter: YNB 1.7 g; Galactose 20.0 g; histidine 0.02 g; leucine 0.1 g; lysine 0.02 g; uracil 0.02 g) was used for detecting the viability of *S. cerevisiae* and its derivatives. *Escherichia coli* was used for propagating plasmids.

### RNA isolation, quantitative real-time PCR and western blotting

Total RNA was isolated using Trizol Reagent (Invitrogen, USA) according to the commercial protocol and digested by DNase I to remove the genomic DNA as described previously [24, 25]. cDNA was obtained using the PrimeScript™ RT Reagent Kit (TaKaRa). Synthesis cDNA and real-time RT-PCR were performed as described previously [24]. Western blot analysis of the isopenicillin N synthase PcbC was performed and the glyceraldehyde-3-phosphate dehydrogenase (AcGapdh, GenBank accession No. MF383617) was used as control [21, 22].

### Identification of *Acatg8* and heterologous complementation of the *S. cerevisiae ATG8* mutant

All primers used in this study were listed in Additional file 1: Table S2. We searched the genomic DNA sequence of *A. chrysogenum* CGMCC 3.3795 using the BLASTX program in the National Center for Biotechnology Information (NCBI). A query sequence, which encodes a putative protein showed 78% identity to Atg8 from *S. cerevisiae*, was designated *Acatg8*. To characterize *Acatg8*, the *A. chrysogenum* wild type strain (WT) was cultured in TSA liquid medium at 28 °C on a rotary shaker (220 rpm) for 48 h. The supernatant was discarded after centrifugation at 12,000 rpm for 5 min. After draining the mycelia with filter paper, liquid nitrogen was added to freeze the mycelia quickly. Then, the mycelia were crushed with sterilized pestle and mortar. DNA Quick Plant System (TianGen, China) and Trizol Reagent were used to isolate the fungal genomic DNA and total RNA respectively. DNA or cDNA of the speculated *Acatg8* gene were amplified with primers Acatg8-F/Acatg8-R and inserted into the vector pEASY-Blunt (TransGen, Beijing) to generate pEB::Acatg8 and pEB::CAcatg8, respectively. The inserts of pEB::Acatg8 and pEB::CAcatg8 were verified by sequencing.

To complement the *S. cerevisiae ATG8* mutant, the cDNA of *Acatg8* was inserted into pYES2 (Invitrogen) under control of the yeast GAL1 promoter. The resulting plasmid pYES2::CAcatg8 was introduced into the *S. cerevisiae ATG8* mutant using a small-scale yeast transformation protocol (Invitrogen, V825-20). Expression of *Acatg8* in the transformants was verified by RT-PCR. Viability of the *S. cerevisiae ATG8* mutant and its complemented strains was detected after incubation for 18 days on the nitrogen-starved medium (SG-N).

### Localization of AcAtg8 in *A. chrysogenum*

The DNA fragment containing PAcatg8-GFP-Acatg8-T was amplified from pCMVPEAT [21] with primers peatF/R. After digestion with *Swa*I, the fragment was ligated into the corresponding sites of pAgB to give pAgB::PAcatg8-GFP-Acatg8-T. Finally, pAgB::PAcatg8-GFP-Acatg8-T was introduced into WT through ATMT. An Axio-observer A1 microscope (Carl Zeiss) was used for microscope observation. Zeiss AxioCam MR camera was used to capture the images of cells. For editing images, AxioVision software and Adobe-Photoshop CS3 software were used.

### Constructions of the *Acatg8* disruption mutant and its complemented strain

To construct the *Acatg8* disruption mutant, a 5480 bp DNA fragment containing *Acatg8* was amplified from WT with primers Acatg8DF/Acatg8DR and inserted into pEASY-Blunt (TransGen, Beijing) to generate pEB::Acatg8LR. After verified by sequencing, pEB::Acatg8LR was digested by *Sal*I. The 1.5 kb *Sal*I DNA fragment containing the bleomycin resistant gene (*ble*) from pJLRNAi was inserted into the corresponding site of pEB::Acatg8LR to give the plasmid pEB::Acatg8LR-B. Then, pEB::Acatg8LR-B was digested by *Swa*I and the DNA fragment containing *Acatg8*-*ble* was inserted into pAg1H3. The resulting plasmid pAg::Acatg8LR-B was introduced into WT via ATMT as described previously [22]. After 3 days of co-incubation at 24 °C, the bleomycin resistant and hygromycin B sensitive transformants were selected as the *Acatg8* disruption mutant (∆Acatg8). Finally, ∆Acatg8 was verified by PCR with gene outside primers Acatg8-outF/R and Southern hybridization.

For genetic complementation, a 2331 bp DNA fragment containing the complete *Acatg8* was amplified by PCR with primers Acatg8C-F/Acatg8C-R and subcloned into the *Swa*Ι site of pAg1H3 to give pAg::Acatg8C. Finally, pAg::Acatg8C was introduced into ∆Acatg8 via ATMT and the transformants were selected in the TSA medium with 50 μg ml^−1^ hygromycin B. The transformants were further verified by RT-PCR. One of them was randomly selected as the complemented strain (Acatg8C) and used in subsequent experiments.

### Inducible expression of *AcAtg8* in ∆Acatg8

For inducible expression of *Acatg8* in ∆Acatg8, a 1.5 kb DNA fragment containing the xylose/xylan-inducible promoter (xyl^P^) was amplified with primers xyl^P^-F/R and inserted into the *Hind*III site of pEASY-Blunt, to give the plasmid pEB::xyl^P^. The *Acatg8* terminator region was amplified with primers Ter-F/R and inserted into the *Bam*HI site of pEASY-Blunt, to give the plasmid pEB::T. The green fluorescent protein (GFP) encoding gene without stop codon was amplified from the plasmid pEGFP-N1 (Clontech) with primers gfpF/gfpNR and inserted into the *Bgl*II site of pEASY-Blunt to give the plasmid pEB::GFP. The *Acatg8* without stop codon was amplified from the cDNA of *A. chrysogenum* with primers atg8F/R and inserted into the *Xba*I site of pEASY-Blunt to give the plasmid pEB::CAcatg8. The xyl^P^, GFP, *Acatg8* and the terminator region were in turn ligated into pCMV3xFlag-10 to generate pCMV::xyl^P^-GFP-Acatg8-T. After digested with *Swa*I, the DNA fragment containing xyl^P^-GFP-Acatg8-T was ligated into the corresponding sites of pAg1H3. Finally, the resulting plasmid pAg::xyl^P^-GFP-Acatg8-T was introduced into ∆Acatg8 through ATMT. For inducible expression of *Acatg8*, the resulting strain ∆Acatg8/pAg::xyl^P^-GFP-Acatg8-T was incubated in the LPE or modified MDFA medium supplemented with 1% xylose.

### Detection of autophagy

Transmission electron microscopy (TEM) was used for detecting the fungal autophagy as described previously [21, 26]. For monodansyl cadavarine (MDC) dye analysis, 1 × 10^6^ conidia of WT, ∆Acatg8 and Acatg8C were added to 20 ml of TSA medium and incubated at 28 °C for 20 h. The mycelia were harvested and washed with sterilized distilled water for three times, and then transferred into distilled water supplemented with 2 mM of phenylmethylsulfonyl fluoride (PMSF). The fungal cultures were collected after 4 h incubation and stained with MDC (Sigma-Aldrich, D-4008) at a final concentration of 60 µM for 30 min in the dark. After rinsed three times with water, samples were observed under fluorescence microscopy.

### Detection of fungal conidiation and cephalosporin C production

Conidiation was detected as described previously [27]. Fermentation of *A. chrysogenum* and detection of CPC production were performed as described previously [21, 28]. *Bacillus subtilis* CGMCC 1.1630 was used as the indicator strain of CPC production.

### Detection of PcbC and CefD2

For detecting expression of *pcbC*, a plasmid containing the PcbC-GFP fusion protein encoding gene was constructed. The *pcbC* coding region and its promoter region were amplified by PCR with primers Ppcbc-F/R and pcbC-F/R, respectively. After verified by sequencing, the amplified fragments were introduced into pCMV-GFP-T to give pCMV::PpcbC-pcbC-GFP-T. For detecting expression of *cefD2*, the same strategy was carried out. The *cefD2* coding region and its promoter region were amplified with primers PcefD2-F/R and cefD2-F/R, respectively. After verified by sequencing, the amplified fragments were introduced into pCMV-GFP-T to give pCMV::PcefD2-GFP-cefD2-T. Then, the fragments containing PpcbC-pcbC-GFP-T and PcefD2-GFP-cefD2-T were amplified from pCMV::PpcbC-pcbC-GFP-T and pCMV::PcefD2-GFP-cefD2-T respectively and inserted into pAgHB to generate pAg::PpcbC-pcbC-GFP-T and pAg::PcefD2-GFP-cefD2-T. Finally, these two plasmids were introduced into WT and ∆Acatg8, respectively. The expressions and localizations of PcbC and CefD2 were observed under fluorescence microscopy.

### Detection of pexophagy and mitophagy

Detection of pexophagy and mitophagy in *A. chrysogenum* was performed as described previously [22].

## Results

### Identification of the *ATG8* homologue *Acatg8* from *A. chrysogenum*

**Fig. 1:**
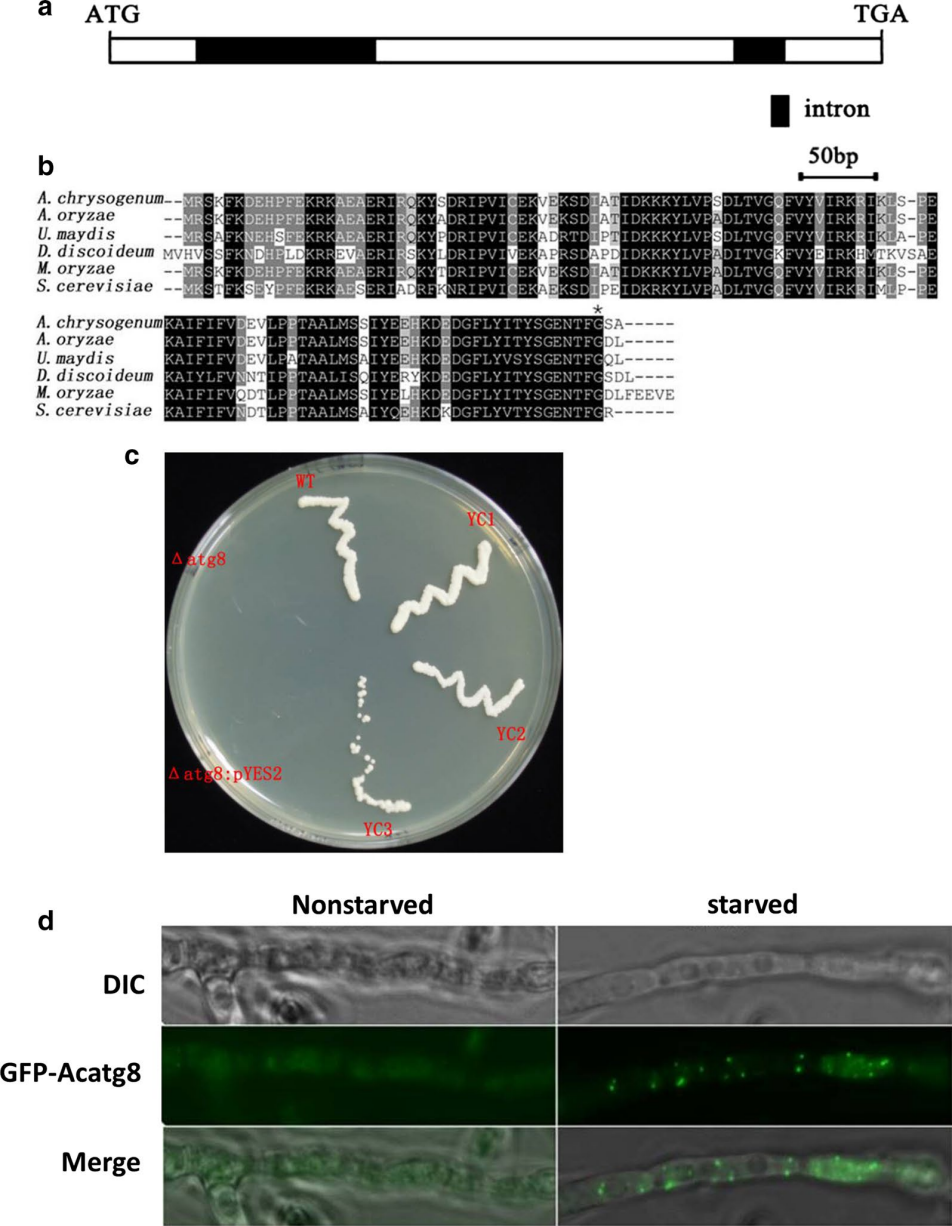
Identification of an *ATG8* gene homologue from *A. chrysogenum*. **a** *Acatg8* with 2 introns. **b** Sequence alignment of AcAtg8 with its homologs. AcAtg8 shows 96% identity to Atg8 from *A. oryzae*, 83% identity to Atg8 from *U. maydis*, 69% identity to Atg8 from *D. discoideum*, 91% identity to Atg8 from *M. oryzae*, 78% identity to Atg8 from *S. cerevisiae*. The asterix indicates the glycine cutting site that is conserved at the C terminal. **c** Viability of ∆atg8 and its complemented strains. The viability of ∆atg8 and its complemented strains was detected after 18 days of incubation on the nitrogen-starved medium (SG-N). **d** Distribution of AcAtg8 in *A. chrysogenum*. Fluorescence observation demonstrated that AcAtg8 was widely distributed throughout the hyphae when the fungal strains grew under nutrient-rich conditions (Nonstarvation), while AcAtg8 was transferred into vacuoles under starvation condition (starvation). WT: the *S. cerevisiae* wild-type strain; ∆atg8: the *S. cerevisiae ATG8* mutant; YC1-3: the complemented strains of ∆atg8; ∆atg8/pYES2: ∆atg8 carrying the plasmid pYES2 as the control; DIC; differential interference contrast; GFP: green fluorescent protein

Since Atg8 proteins are highly conserved in fungi [29], an open reading frame (ORF) encoding an Atg8 homologous protein was identified in *A. chrysogenum* and it was designated *Acatg8* (GenBank accession No. KJ569771). The cDNA of *Acatg8* was amplified and sequenced. Comparing the sequences of *Acatg8* and its cDNA, two introns (localized in positions + 93 to + 284 and + 504 to + 566 with respect to the translation initiation site) were found (Fig. 1a). The deduced protein of *Acatg8* contains 118 amino acids, and its theoretical molecular weight is 13.7 kDa. Based on sequence alignment, AcAtg8 shows 96% identity to Atg8 from *A. oryzae*, 83% identity to Atg8 from *Ustilago maydis*, 69% identity to Atg8 from *Dictyostelium discoideum*, 91% identity to Atg8 from *M. oryzae*, 78% identity to Atg8 from *S. cerevisiae* (Fig. 1b).

To address whether AcAtg8 has the same physiological function in autophagic process as Atg8 of *S. cerevisiae*, the plasmid pYES2::Acatg8 was constructed and introduced into the *S. cerevisiae ATG8* mutant (∆atg8) which generally dies after a long time incubation under starvation condition. Three heterologous complemented strains of ∆atg8, designated as YC1-3, were obtained through uracil selection and *Acatg8* expression was confirmed by RT-PCR (Additional file 1: Fig. S1). After 18 days of incubation on the nitrogen-starved medium (SG-N) at 30 °C, the *S. cerevisiae* wild-type strain (WT), ∆atg8 and YC1-3 were shifted to YPD medium. Both WT and YC1-3 grew well, but ∆atg8 and ∆atg8/pYES2 could not grow due to a long time nitrogen starvation (Fig. 1c), indicating that *AcAtg8* complements the *atg8* mutation in *S. cerevisiae*. Combined with the sequence alignment analysis, *Acatg8* is the homologous gene of *S. cerevisiae ATG8*.

### *Acatg8* is essential for the autophagic process of *A. chrysogenum*

To detect the distribution of AcAtg8 in *A. chrysogenum*, pAgB::GFP-AcAtg8 used for *GFP*-*Acatg8* expression was constructed and introduced into WT. The verified transformant was incubated in TSA medium for 16 h and then shifted into the sterile water containing 2 mM of phenylmethylsulfonyl fluoride (PMSF) for additional 4 h incubation. AcAtg8 was widely distributed throughout the hyphae under nutrient-rich growth conditions (Nonstarvation), while it was punctually localized under starvation growth conditions (starvation) (Fig. 1d). The distribution of AcAtg8 in *A. chrysogenum* resembles that of Atg8 in *S. cerevisiae* [30].

**Fig. 2:**
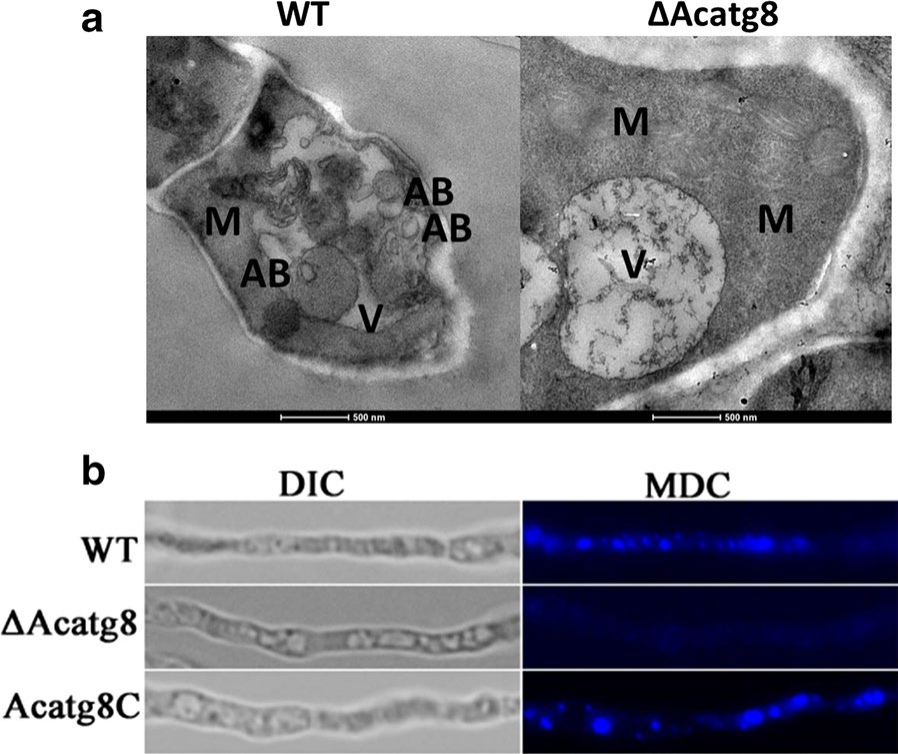
Effect of *Acatg8* deficiency on autophagy of *A. chrysogenum*. **a** Transmission electron microscopy (TEM) analysis of WT and ∆Acatg8 under nutrition-shift condition. The autophagosomes were observed in the vacuole of WT, but not in that of ΔAcatg8 under starvation condition. V: vacuole; AB: autophagic body; M: mitochondria. Bars, 500 nm. **b** Distribution of autophagosomes in WT, ∆Acatg8 and Acatg8C. The autophagosomes were observed in the vacuoles of WT, but not in the vacuoles of ∆Acatg8. The complemented strain restored the wild-type phenomenon. Images were observed after 4 h induction. DIC: differential interference contrast; MDC: monodansyl cadavarine

To further address its function, *Acatg8* was disrupted in *A. chrysogenum* via homologous recombination (Additional file 1: Fig. S2). The *Acatg8* disruption mutant (∆Acatg8) was verified by PCR and Southern hybridization, respectively (Additional file 1: Fig. S2). After transferred into the starvation condition, WT,∆Acatg8 and the complemented strain (Acatg8C) were cultured for 4 h. Transmission electron microscopy (TEM) demonstrated that autophagosomes were localized in the vacuoles of WT but not in ∆Acatg8, indicating that disruption of *Acatg8* inhibits autophagic process of *A. chrysogenum* (Fig. 2a). In consistent with TEM observation, monodansyl cadavarine (MDC) analysis demonstrated that autophagosomes were localized in the vacuoles of WT, but not in the vacuoles of ΔAcatg8 (Fig. 2b). Acatg8C restored the wild-type phenomenon.

### Growth, conidial germination and conidiation were reduced in ∆Acatg8

**Fig. 3:**
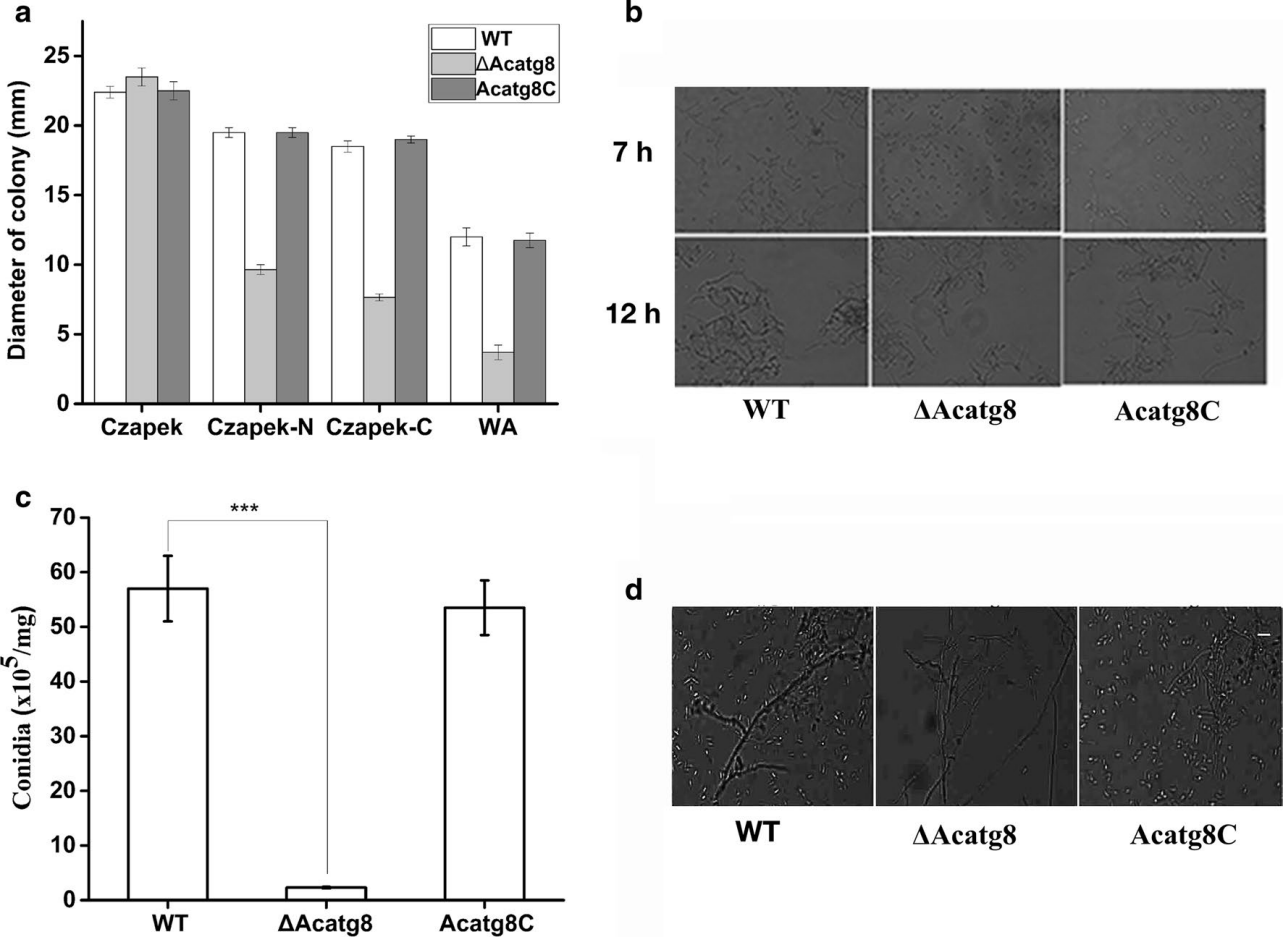
Growth, conidial germination and conidiation of WT, ∆Acatg8 and Acatg8C. **a** Growth of WT, ∆Acatg8 and Acatg8C was detected. After incubated in TSA medium for 72 h, hyphae of WT, ∆Acatg8 and Acatg8C were transferred to Czapek medium, Czapek–N medium (No nitrogen), Czapek–C medium (No carbon) and WA medium (water with agar 1%). The colony diameters of different strains were measured. The asterix indicates that the differences between strains are significant. p < 0.001***; p < 0.01**; p < 0.05*. **b** Disruption of *Acatg8* reduces conidial germination. When grown on LPE medium, the conidial germination of ∆Acatg8 was delayed compared with that of WT and Acatg8C. **c** Disruption of *Acatg8* reduces fungal conidiation. The number of conidia in ∆Acatg8 grown on LPE medium for 7 days was only about 5% of that in WT. Error bars show standard deviations of three independent experiments. The asterix indicates that the differences between strains are significant. p < 0.001***; p < 0.01**; p < 0.05*. **d** Conidiation of WT, ∆Acatg8 and Acatg8C on LPE medium for 7 days was observed under microscope

Since *ATG8* is essential for yeast survival under starvation condition, the effect of *Acatg8* deficiency on the growth of *A. chrysogenum* was detected under nutrient-starvation condition. After grown on TSA medium for 3 days, the hyphae of WT, ∆Acatg8 and Acatg8C were collected and transferred into Czapek medium, Czapek–N medium (No nitrogen), Czapek–C medium (No carbon) and WA medium (water with agar 1%) respectively and incubated for additional 7 days. Comparing the colonial diameters of different strains demonstrated that ∆Acatg8 grew slower than WT and Acatg8C, and ∆Acatg8 hardly grew on WA medium (Fig. 3a). These results indicated that *Acatg8* is important for the growth of *A. chrysogenum* under starvation condition.

When grown on LPE medium, spore germination of ∆Acatg8 was delayed compared with that of WT and Acatg8C (Fig. 3b). It is possible that autophagy is also involved in spore germination of *A. chrysogenum.* The plasmid pAg::PAcatg8-GFP-Acatg8-T used for *GFP*-*Acatg8* expression was constructed and introduced into WT, and germination of the verified transformant was detected under fluorescence microscope. Punctates of GFP-AcAtg8 were observed during spore germination, implying autophagosomes were formed (Additional file 1: Fig. S3). It is speculated that fungal cells degrade the stored nutrients (glycogen, fat droplets, etc.) through the autophagic process when the spores rapidly germinate under appropriate conditions. Similar with that found in *M. oryzae* [31], disruption of *Acatg8* dramatically reduced conidiation of *A. chrysogenum* (Fig. 3c and 3d). Meanwhile, the transcript levels of *AcbrlA, AcwetA* and *AcabaA*, as the key genes for conidiation of *A. chrysogenum* [27], were evaluated. A non-conidiation related gene glyceraldehyde-3-phosphate dehydrogenase encoding gene *AcGapdh* (GenBank accession No. MF383617) of *A. chrysogenum* was used as control. Transcriptional analysis revealed that disruption of *Acatg8* significantly decreased the transcriptional level of *AcbrlA,AcwetA* and *AcabaA* (Additional file 1: Fig. S4). These results indicated that *Acatg8* is important not only for the growth and conidial germination, but also for the fungal conidiation of *A. chrysogenum.*

### Addition of exogenous carbon sources partially restores the conidiation of ∆Acatg8

Combined with the results above, we speculated that ∆Acatg8 could not recycle its own cellular components due to autophagic defect. Therefore, ∆Acatg8 could not provide enough nutrients or energy for conidiation. To verify our speculation, exogenous nitrogen and carbon sources were added in LPE medium. As expected, supplementation of exogenous carbon sources partially restored the conidiation of ∆Acatg8 (Table 1). However, supplementation of exogenous nitrogen sources could not restore the conidiation of ∆Acatg8 (Table 1). It has been reported that the conidial formation was affected by the cellular glycogen in *M. oryzae* [31]. In *M. oryzae*, disruption of *MoATG8* dramatically reduced the conidial formation and exogenous supply of glucose or deletion of a glycogen phosphorylase Gph1 could suppress the conidiation defects, indicating glycogen homeostasis is important for fungal conidiation. Like in *M. oryzae*, carbon source such as glucose in the culture medium may affect glycogen homeostasis or the enzymes involved in glycogen metabolism, and in turn partially recover the phenotype caused by autophagic defect in ∆Acatg8.

**Table 1:** Effects of supplemented carbon/nitrogen sources on conidiation of *A. chrysogenum*

Supplemented sources	Conidia (×10^7^/plate)		
	WT	∆Acatg8	Acatg8C
Carbon sources			
CK	13.92	0.98**	13.85
Glucose	13.49	7.75*	12.51
Sucrose	12.06	7.62*	12.96
Inositol	11.75	5.86*	10.95
Mannitol	10.65	6.86*	11.92
Nitrogen sources			
CK	13.92	0.98**	13.85
NH_4_Cl	5.96	0.89**	5.26
NaNO_3_	4.75	0.85**	4.47
NH_4_NO_2_	2.82*	1.07**	3.73
Met	2.94*	1.97*	2.88
Pro	3.84	1.63**	2.76

Conidia formation in WT, ∆Acatg8 and Acatg8C grown on the LPE medium supplemented with different carbon/nitrogen sources. Glucose, sucrose, inositol or mannitol was supplemented at the final concentration of 1%. Ammonium chloride (NH_4_Cl), niter nitrate (NaNO_3_), ammonium nitrate (NH_4_NO_2_), methionine (Met) or proline (Pro) was added in the LPE medium at the final concentration of 1%. Numbers of conidia were counted after 7 days incubation. CK, the medium without supplementation of carbon/nitrogen sources. The data are derived from the average of three independent experiments. The asterix indicates that the differences between strains are significant. p < 0.001 ***; p < 0.01 **; p < 0.05 *

### Cephalosporin C production is significantly increased in ∆Acatg8

**Fig. 4:**
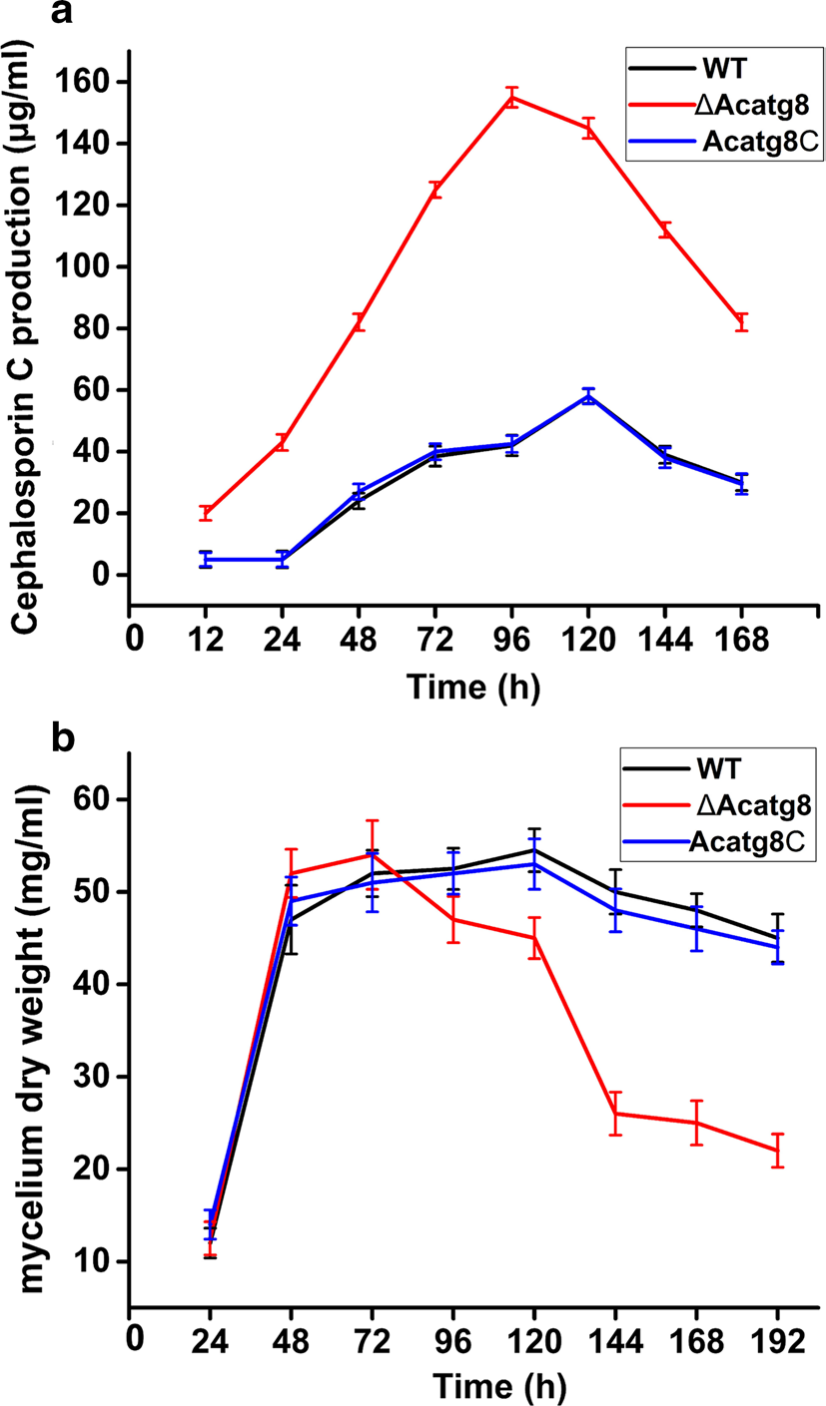
Disruption of *Acatg8* significantly increases cephalosporin C production of *A. chrysogenum*. **a** Cephalosporin C production of WT, ∆Acatg8 and Acatg8C was detected during fermentation in the modified MDFA medium. **b** Biomass of WT, ∆Acatg8 and Acatg8C during fermentation. The fungal mycelium dry weight was determined after drying at 42 °C in a hot air oven until a constant weight. After 72 h fermentation, the mycelium dry weight of ∆Acatg8 dropped quickly. Error bars show standard deviations of three independent experiments

**Fig. 5:**
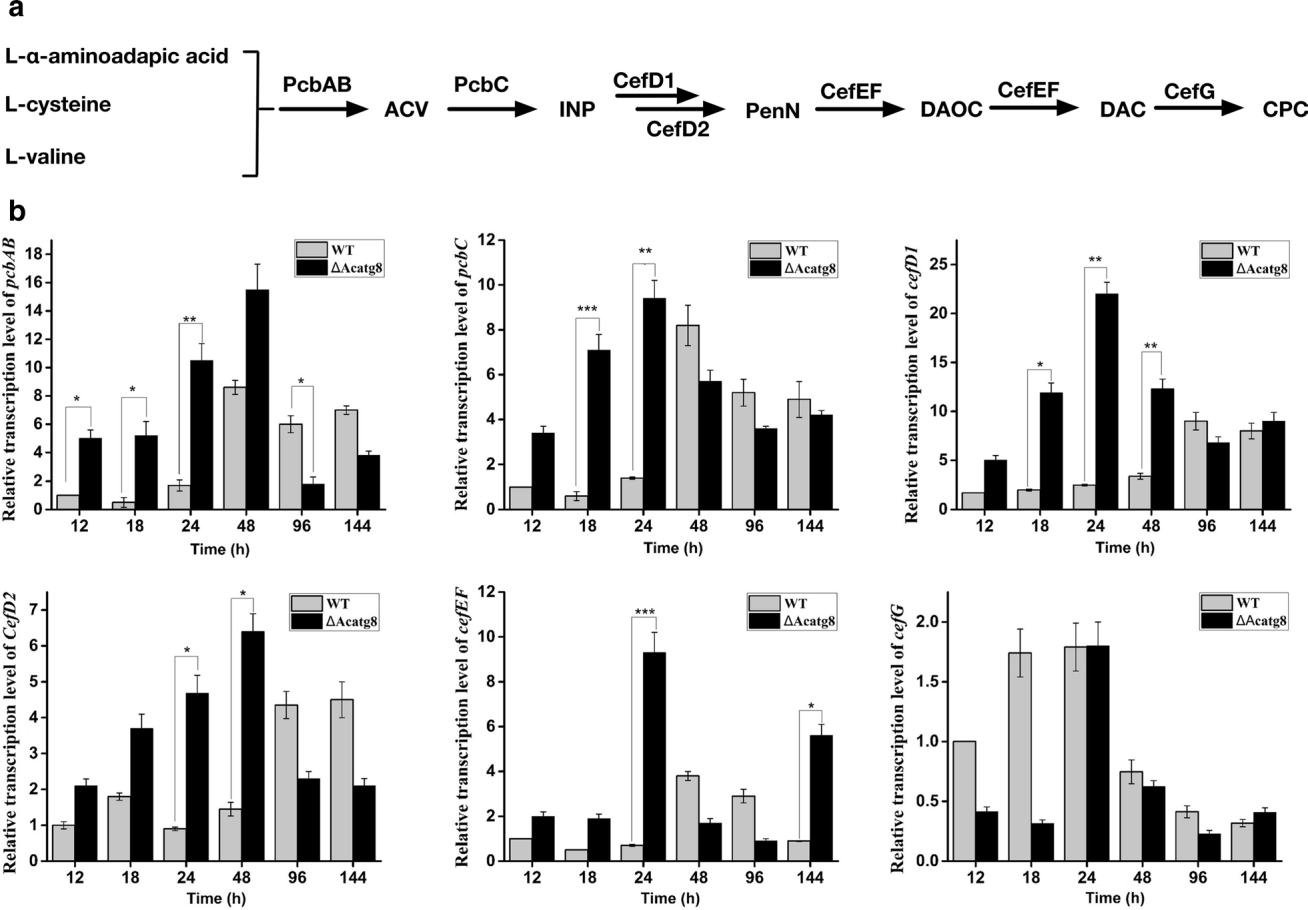
Transcriptional analysis of the cephalosporin biosynthetic genes. **a** The cephalosporin C biosynthetic pathway. ACV, tripeptide δ-(l-α-aminoadipyl)-l-cysteinyl-d-valine; IPN: isopenicillin N; PenN: Penicillin N; DAOC: deacetoxycephalosporin C; DAC: deacetylcephalosporin C; CPC: cephalosporin C. **b** The relative transcriptional level of the cephalosporin biosynthetic genes. The relative abundance of mRNAs was standardized against the level of *actin* gene. The gray columns and black columns represent the relative gene transcriptions of WT and ∆Acatg8, respectively. Error bars show standard deviations of three independent experiments. The asterix indicates that the differences between strains are significant. p < 0.001***; p < 0.01**; p < 0.05*

Our previous study showed that disruption of *Acatg1* increased CPC yield through reducing degradation of cephalosporin biosynthetic proteins [21], indicating deficiency of autophagy facilitates antibiotic production. Therefore, the CPC production of ∆Acatg8 was detected. As expected, the CPC production increased threefold in ∆Acatg8 compared with that in WT (Fig. 4a). What excites us most is that not only the yield of CPC was increased, but also CPC was produced earlier in ∆Acatg8. Only 12 h fermentation, ∆Acatg8 started to produce appreciable quantity of CPC in MDFA medium. While the CPC production was hardly detected in WT before 48 h fermentation. Unfortunately, disruption of *Acatg8* significantly decreased the fungal biomass (Fig. 4b). Furthermore, transcript levels of the key genes (*pcbAB, pcbC*, *cefD1, cefD2*, *cefEF* and *cefG*) for cephalosporin biosynthesis in *A. chrysogenum* were measured by real time RT-PCR (Fig. 5). In consistence with CPC production, the transcription of these key genes was maintained continuously at a relatively high level in ∆Acatg8 from 12 to 48 h. These results indicated that at least one of the reasons for the yield increase and earlier production of CPC was due to the elevated expression of cephalosporin biosynthetic genes. However, the transcription of these key genes in ∆Acatg8 was quickly declined after 96 h fermentation and the transcription level was even lower than that in WT. To explain the contradiction between CPC yield and the biosynthetic gene transcripts at the late stage of fermentation, the degradation of PcbC was detected in ∆Acatg8 and WT.

**Fig. 6:**
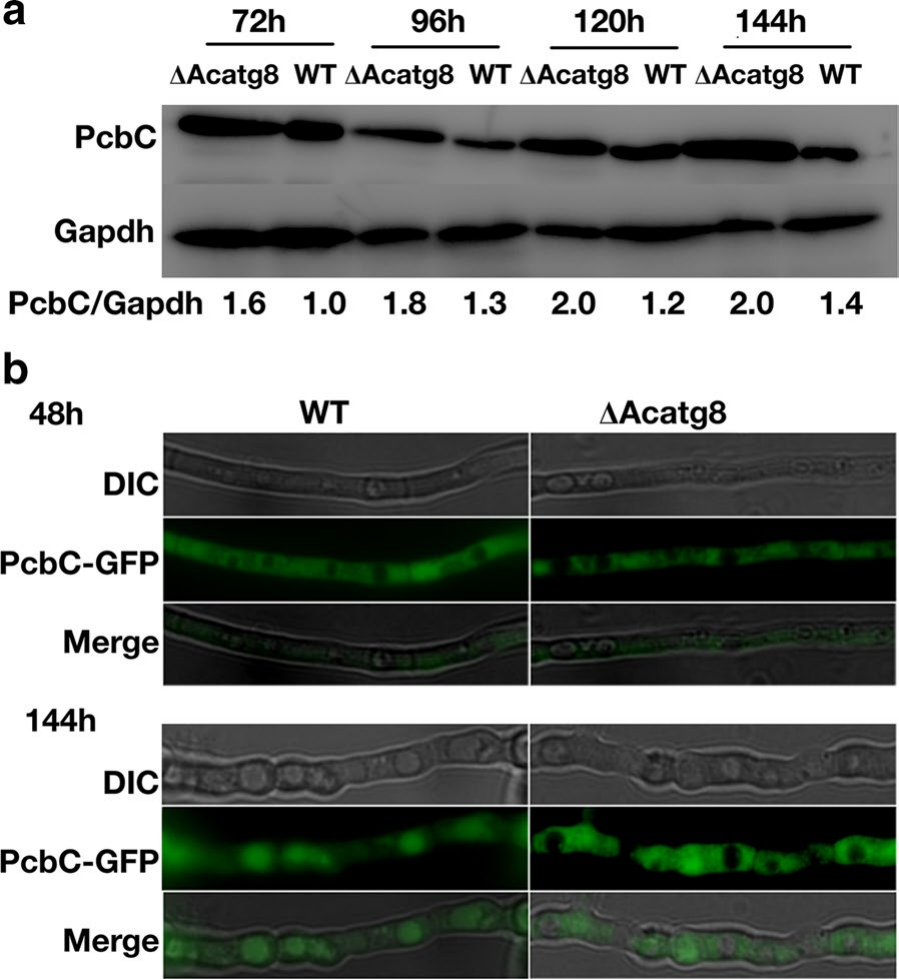
Detection of PcbC in WT and ∆Acatg8 during fermentation. **a** Western blot analysis of PcbC in WT and ∆Acatg8. Mycelia cultured in the modified MDFA medium for 72, 96, 120 and 144 h were collected. 50 μg of total protein was loaded for Western blot analysis with anti-PcbC. Gapdh was used as control. The ratio of PcbC to Gapdh is shown at the bottom. **b** PcbC was detected under fluorescence microscope after 48 and 144 h fermentation. PcbC in WT and ∆Acatg8 were labeled with a green fluorescent protein (GFP)

Western blot analysis revealed that the quantity of PcbC in ∆Acatg8 was higher than that in WT not only at the early stage but also at the late stage of fermentation (Fig. 6a), suggesting accumulation of PcbC is one of the reasons for cephalosporin yield increment in ∆Acatg8. Fluorescence observation further demonstrated that there was no fluorescence in the vacuoles of WT and ∆Acatg8 at the early stage of fermentation. At the late stage of fermentation, the vacuoles of WT were filled with fluorescence, indicating most of the PcbC was transferred into vacuoles for degradation. While there was no fluorescence in the vacuoles of ∆Acatg8 and PcbC was accumulated in the cytoplasm (Fig. 6b), indicating PcbC was retained in ∆Acatg8. Therefore, it is possible that deficiency of autophagy reduces the degradation of cephalosporin biosynthetic proteins and in turn increases CPC production at the late stage of fermentation in *A. chrysogenum*.

### Disruption of *Acatg8* dramatically reduces fungal viability at the late stage of fermentation

It is interesting to find that disruption of *Acatg8* remarkably increased CPC production. However, the fungal biomass demonstrated the viability of ∆Acatg8 was significantly reduced especially at the late stage of fermentation (Fig. 4b). These results further indicated that *Acatg8* is very important for fungal survival and related with CPC production. It is reasonable that WT can degrade cellular components and recycle nutrient through autophagy at the late stage of fermentation, while ∆Acatg8 could not since its autophagic process is inhibited. In addition, many toxic substances especially reactive oxygen species (ROS) were gradually accumulated along with the metabolic process during extension of cell survival time. The accumulation of ROS is speculated as an important factor for causing the death of ∆Acatg8. To confirm our speculation, the citric acid dehydrogenase (Cit) fused with GFP was used to mark mitochondria (Additional file 1: Fig. S5). The fluorescence observation showed that mitochondria of WT were almost degraded in vacuoles at the late stage of fermentation, while no fluorescence was observed in vacuoles of ∆Acatg8 and a large number of mitochondria were accumulated in the cytoplasm of ∆Acatg8. Thus, disruption of *Acatg8* not only caused autophagic defect but also blocked the normal degradation of mitochondria. The accumulation of mitochondria, especially those dysfunctional mitochondria, could lead to the accumulation of ROS which causes premature death of ∆Acatg8.

### Inducible expression of *Acatg8* in ∆Acatg8 improves fungal viability but maintains high cephalosporin C yield at the late stage of fermentation

The premature death is unfavorable for CPC production, especially in industry. To overcome the premature death of ∆Acatg8, the endogenous xylose/xylan-inducible promoter xyl^P^ was used for inducible expression of *Acatg8*. The promoter xyl^P^, which is induced by xylose/xylan but repressed by glucose, has been successfully used in *A. chrysogenum* [32]. The plasmid pAg::xyl^P^-GFP-Acatg8-T was constructed and introduced into ∆Acatg8 (Additional file 1: Fig. S6). As expected, ∆Acatg8/pAg::xyl^P^-GFP-Acatg8-T dramatically increased fungal conidial formation in LPE plates supplemented with 1% xylose. The number of conidia in ∆Acatg8/pAg::xyl^P^-GFP-Acatg8-T was about the same as that in WT (Additional file 1: Fig. S6). Then the expression of *GFP* in ∆Acatg8/pAg::xyl^P^-GFP-Acatg8-T was detected at the sixth day of fermentation. Fluorescence observation indicated that *GFP* was expressed under control of xyl^P^ at the late stage of fermentation when glucose in the medium was depleted (Additional file 1: Fig. S7). In consistent with the expression of *GFP*, *Acatg8* was expressed in the presence of xylose when the non-inducing carbon source glucose was depleted in ∆Acatg8/pAg::xyl^P^-GFP-Acatg8-T.

**Fig. 7:**
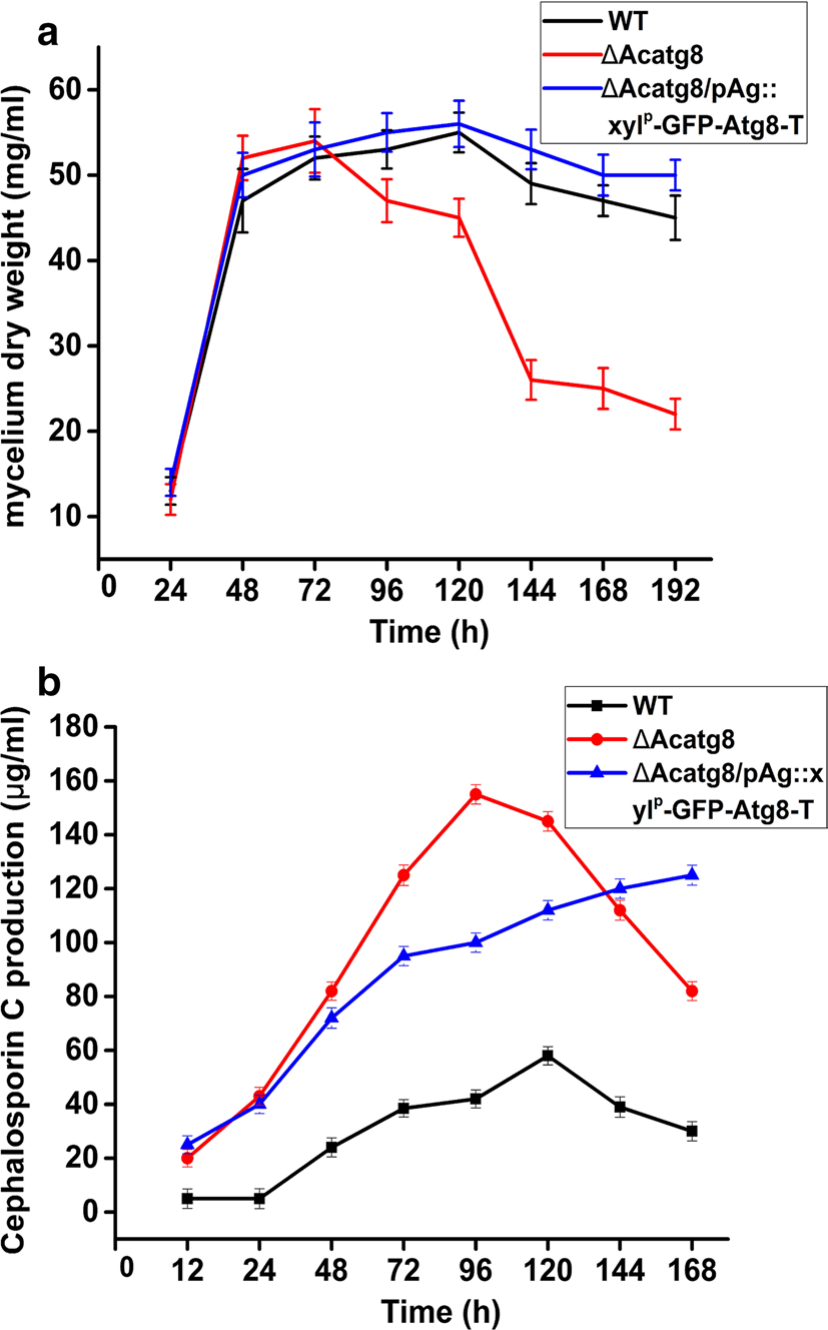
Cephalosporin C production of WT, ∆Acatg8 and ∆Acatg8/xyl^P^-GFP-Atg8 in the modified MDFA medium supplemented with 1% xylose. **a** Growth of WT, ∆Acatg8 and ∆Acatg8/pAg::xyl^P^-GFP-Acatg8-T during fermentation. The fungal mycelium dry weight was determined after drying at 42 °C in a hot air oven until a constant weight. ∆Acatg8/pAg::xyl^P^-GFP-Acatg8-T, ∆Acatg8 was complemented with *Acatg8* under the xylose inducible promoter xyl^P^. **b** Cephalosporin C production of WT, ∆Acatg8 and ∆Acatg8/pAg::xyl^P^-GFP-Acatg8-T. Cephalosporin C production was determined by bioassays against *B. subtilis* CGMCC 1.1630. 40 μl of culture filtrates of fermentation was used to detect the cephalosporin C production. The plate was added 50,000 units of penicillinase to exclude penicillin in culture filtrates. Error bars show standard deviations of three independent experiments

As mentioned above, the biomass of ∆Acatg8 was only about 60% of WT at the late stage of fermentation due to the autophagic defect. When *Acatg8* was expressed in ∆Acatg8/pAg::xyl^P^-GFP-Acatg8-T, the fungal viability was restored to the wild-type level just like the conidial formation (Fig. 7a). The CPC production of ∆Acatg8/pAg::xyl^P^-GFP-Acatg8-T was also detected. Like ∆Acatg8, ∆Acatg8/pAg::xyl^P^-GFP-Acatg8-T still kept the ability to produce more CPC in presence of xylose (Fig. 7b). Although it was lower than that of ∆Acatg8 at 96 h fermentation, the CPC yield of ∆Acatg8/pAg::xyl^P^-GFP-Acatg8-T maintained at the high level during prolonged fermentation.

## Discussion

Autophagy is the highly conserved eukaryotic physiological process which plays a vital role in maintaining intracellular carbon and nitrogen homeostasis [33]. Previous study showed that autophagy was related with morphological differentiation and antibiotic production in filamentous fungi [15, 21]. During fermentation, the morphological differentiation of the high CPC producing strain proceeded rapidly. Swollen hyphal fragments and arthrospores appeared at 72 h fermentation [34]. In contrast with the arthrospore formation, an inverse relation was found between the fungal growth and CPC production [35]. Combining our results that inhibition of autophagy increased CPC production but decreased the fungal viability, it is possible that the autophagy was reduced in the high CPC producing strain.

In this study, the physiological function of core autophagy-related gene *Acatg8* was investigated. Disruption of *Acatg8* resulted in a significant increment of CPC yield. However, the fungal viability and conidiation were remarkably reduced in ∆Acatg8 due to the autophagic defect. To overcome the defects of the fungal viability and conidiation, the xylose/xylan-inducible promoter xyl^P^ was used to control the expression of *Acatg8* in ∆Acatg8. Through inducible expression of *Acatg8* in ∆Acatg8, the fungal conidiation and growth were restored to the wild-type level, while the CPC production still maintained at a high level as we expected.

Peroxisomes are single-membrane organelles not only involved in the β-oxidation of fatty acids but also involved in secondary metabolite biosynthesis [36]. In *P. chrysogenum*, the final steps for conversion of isopenicillin N to penicillin G occur in peroxisomes [37, 38]. It was also found that the high penicillin producing strains contain more peroxisomes and increasing peroxisome numbers by overexpression of *pex11* increased penicillin production 2–3 fold [39, 40]. Sequence analysis revealed that some essential proteins for cephalosporin biosynthesis contain putative peroxisomal targeting signals (PTS), indicating that peroxisomes are also involved in CPC production [41]. It is reasonable that not only enhancing peroxisome proliferation but also inhibiting peroxisome degradation could increase peroxisome numbers. Disruption of *Acatg8* clearly inhibited peroxisome degradation and increased peroxisome accumulation (Additional file 1: Fig. S8). Since CefD2 is localized in peroxisomes, increase peroxisome numbers could enhance the concentration of CefD2. As expected, fluorescence observation demonstrated CefD2 retained in ∆Acatg8 even after 144 h fermentation (Additional file 1: Fig. S9). We speculate that peroxisome accumulation is one of the main reasons for the CPC enhancement in ∆Acatg8. However, the peroxisome accumulation through disruption of *Acatg11* did not increase CPC production [22]. It is possible that *Acatg11* has multiple functions and some of them are positive related with CPC production.

In *S. cerevisiae*, autophagy is the main mechanism for maintaining cellular survival under starvation condition [42, 43]. Fungal cells tend to die once autophagy is inhibited [44, 45]. In filamentous fungi, autophagy is used for recycling the carbon and nitrogen sources and reconstituting the intracellular components during fungal morphological differentiation. Under starvation condition, the mycelia base cells will degrade their material and transport to the apical cells. These nutrients were used to promote the mycelial growth [46]. This mechanism allows the colony to expand and makes it easier to find usable substances. Like most of filamentous fungi, the disruption mutant of *Acatg8* could not grow well under starvation condition due to the autophagic defect, indicating that *Acatg8* is necessary for the survival of *A. chrysogenum* under starvation condition.

A large number of accumulated mitochondria were also observed in ∆Acatg8 at the late stage of fermentation (Additional file 1: Fig. S5). In *S. cerevisiae*, autophagic defect leads to mitochondrial dysfunction and accumulates excessive ROS [47]. In mammalian cells, excessive ROS will induce the autophagy-mediated cell death [48]. This could be the main reason of ∆Acatg8 viability reduction since dysfunctional mitochondria produce excessive ROS which impairs fungal survival.

Premature death of ∆Acatg8 is an intractable problem since it will result in cephalosporin biosynthesis termination, it was also found in the *Acatg1* disruption mutant [21]. To solve this problem, inducible expression of *Acatg8* under xyl^P^ was performed in ∆Acatg8. Inducible expression of *Acatg8* remarkably improved the viability of ∆Acatg8, especially at the late stage of fermentation when glucose was depleted, while the CPC yield still maintained at a high level. Thus, the inducible expression of autophagy-related genes could be a general method for increasing antibiotic production and maintaining the fungal viability in the autophagic deficiency strains.

## Conclusions

In this study, we identified and characterized an autophagy related gene *Acatg8* which could complement the *ATG8* disruption mutant (Δatg8) of *S. cerevisiae*. AcAtg8 is localized in the cytoplasm and autophagosome of *A. chrysogenum* based on the observation of fluorescently labeled AcAtg8, and the expression of *Acatg8* was clearly induced by starvation. Disruption of *Acatg8* inhibited the autophagosome formation of *A. chrysogenum* and reduced the fungal conidiation, but increased the CPC production through enhancing the transcription of cephalosporin biosynthetic genes and retaining their products. However, disruption of *Acatg8* seriously reduced the fungal viability. Through inducible expression of *Acatg8* under the xylose/xylan-inducible promoter xyl^P^, the fungal viability was restored while the CPC production still maintained at a high level. This study provides a promising approach for antibiotic production improvement through modulating the autophagic process of *A. chrysogenum* and extends our understanding of the relationship between secondary metabolite production and fungal autophagy.

## Additional file

**Additional file 1: Table S1.** Strains and plasmids used in this study. **Table S2.** Primers used in this study. **Fig. S1.** Verification of the heterologous complemented strains of ∆atg8 by RT-PCR. **Fig. S2.** Construction of the *Acatg8* disruption mutant. **Fig. S3.** Localization of AcAtg8 during conidial germination of *A. chrysogenum*. **Fig. S4.** Relative transcriptional level of *AcbrlA, AcwetA* and *AcabaA* for conidiation in WT, ∆Acatg8 and Acatg8C. **Fig. S5.** Degradation of mitochondria in WT and ∆Acatg8. **Fig. S6.** Complementation of ∆Acatg8 with *Acatg8* under control of xyl^P^. **Fig. S7.** Inducible expression of *Acatg8* under control of xyl^P^. **Fig. S8.** Degradation of peroxisomes in WT and ∆Acatg8 during fermentation. **Fig. S9.** Degradation of CefD2 in WT and ∆Acatg8 during fermentation.

## Abbreviations

WT: the wild-type strain
∆Acatg8: the *Acatg8* disruption mutant
Acatg8C: the complemented strain of ∆Acatg8
ACV: tripeptide δ-(l-α-aminoadipyl)-l-cysteinyl-d-valine
IPNS: isopenicillin N synthetase
IPN: isopenicillin N
DAOC: deacetoxycephalosporin C
DAC: deacetylcephalosporin C
ORF: open reading frame
SG-N: nitrogen-starved medium
TEM: transmission electron microscopy
ROS: reactive oxygen species
RFP: red fluorescent protein
GFP: green fluorescent protein
*hph*: hygromycin phosphotransferase gene
*ble*: bleomycin resistance gene
ATMT: *Agrobacterium tumefaciens*-mediated transformation
CPC: cephalosporin C
MDC: monodansyl cadavarine
PMSF: phenylmethylsulfonyl fluoride
