# Supplementary material for: Enhancing the production of cephalosporin C through modulating the autophagic process of Acremonium chrysogenum
Source: Microb Cell Fact. 2018 Nov 13;17:175. doi: 10.1186/s12934-018-1021-9 (PMC6233533; doi:10.1186/s12934-018-1021-9)
Supplement: Supplementary file 1 — Additional file 1: Table S1. Strains and plasmids used in this study. Table S2. Primers used in this study. Fig. S1. Verification of the heterologous complemented strains of ∆atg8 by RT-PCR. Fig. S2. Construction of the Acatg8 disruption mutant. Fig. S3. Localization of AcAtg8 during conidial germination of A. chrysogenum. Fig. S4. Relative transcriptional level of AcbrlA, AcwetA and AcabaA for conidiation in WT, ∆Acatg8 and Acatg8C. Fig. S5. Degradation of mitochondria in WT and ∆Acatg8. Fig. S6. Complementation of ∆Acatg8 with Acatg8 under control of xylP. Fig. S7. Inducible expression of Acatg8 under control of xylP. Fig. S8. Degradation of peroxisomes in WT and ∆Acatg8 during fermentation. Fig. S9. Degradation of CefD2 in WT and ∆Acatg8 during fermentation. [file 12934_2018_1021_MOESM1_ESM.doc]

**Additional file 1**

**Enhancing the production of cephalosporin C through modulating the autophagic process of *Acremonium chrysogenum***

Honghua Li^1,2⊥^, Pengjie Hu^1⊥^, Ying Wang^1⊥^, Yuanyuan Pan^1^ and Gang Liu^1,2*^

^1^ State Key Laboratory of Mycology, Institute of Microbiology, Chinese Academy of Sciences, Beijing 100101, China

^2^ University of Chinese Academy of Sciences, Beijing 100049, China

Email addresses of all authors:

Honghua Li, Email: [huahappy19921208@163.com](mailto:huahappy19921208@163.com); Pengjie Hu, Email: [hpj@mail.ustc.edu.cn](mailto:hpj@mail.ustc.edu.cn); Ying Wang, Email: [wangying.2006h@163.com](mailto:wangying.2006h@163.com); Yuanyuan Pan, Email: panyy@im.ac.cn; Gang Liu, Email: [liug@im.ac.cn](mailto:liug@im.ac.cn).

^⊥^ Contributed equally to this work

* Corresponding author

**Content**

**Table S1** Strains and plasmids used in this study.

**Table S2** Primers used in this study.

**Fig. S1** Verification of the heterologous complemented strains of Δatg8 by RT-PCR.

**Fig. S2** Construction of the *Acatg8* disruption mutant.

**Fig. S3** Localization of AcAtg8 during conidial germination of *A. chrysogenum*.

**Fig. S4** Relative transcriptional level of *AcbrlA, AcwetA* and *AcabaA* for conidiation in WT, ∆Acatg8 and Acatg8C.

**Fig. S5** Degradation of mitochondria in WT and ΔAcatg8.

**Fig. S6** Complementation of ∆Acatg8 with *Acatg8* under control of xyl^P^.

**Fig. S7** Inducible expression of *Acatg8* under control of xyl^P^.

**Fig. S8** Degradation of peroxisomes in WT and ΔAcatg8 during fermentation.

**Fig. S9** Degradation of CefD2 in WT and ΔAcatg8 during fermentation.

**Table S1 Strains and plasmids used in this study.**

| Strains or plasmids | characteristics | Source |
| --- | --- | --- |
| **Strains** |  |  |
| *Acremonium chrysogenum* 3.3795 | The wild-type strain | CGMCC^a^ |
| ΔAcatg8 | The *Acatg8* disruption mutant of *A. chrysogenum* | This study |
| Acatg8C | The complemented strain of ΔAcatg8 | This study |
| WT/GFP -Acatg8 | For the expression of *GFP-Acatg8* in WT | This study |
| ΔAcatg8/GFP-Acatg8 | For the expression of *GFP-Acatg8* in ΔAcatg8 | This study |
| WT/PcbC-GFP | For the expression of PcbC-GFP in WT | This study |
| ΔAcatg8/PcbC-GFP | For the expression of PcbC-GFP in ΔAcatg8 | This study |
| WT/GFP–CefD2 | For the expression of GFP–CefD2 in WT | This study |
| WT/GFP-SKL | Used for detection of peroxisomes in WT | This study |
| ΔAcatg8/GFP-SKL | Used for detection of peroxisomes in ΔAcatg8 | This study |
| WT/cit-GFP | Used for detection of mitochondria in WT | This study |
| ΔAcatg8/cit-GFP | Used for detection of mitochondria in ΔAcatg8 | This study |
| *Saccharomyces cerevisiae* BY4742 | The wild-type strain of yeast | Invitrogen |
| Δatg8 | The *ATG8* disruption mutant of *S. cerevisiae* | Invitrogen |
| YC1-3 | The complemented strain of Δatg8 by *Acatg8* | This study |
| Δatg8/pYES2 | Used for the negative control | This study |
| ΔAcatg8/pAg::xyl^P^-GFP-Acatg8-T | Used for inducible expression of *Acatg8* in ΔAcatg8 | This study |
| *Escherichia coli* DH5α | Used for routine cloning | Gibco BRL |
| *Bacillus subtilis* 1.1630 | Used for detection of cephalosporin C production | CGMCC^a^ |
| *Agrobacterium tumefaciens* AGL-1 | Used for fungal transformation | [1] |
| **Plasmids** |  |  |
| pEASY-Blunt | Routine cloning vector | Transgen |
| pEB::Acatg8 | The *Acatg8* sequence was inserted into pEASY-Blunt | This study |
| pEB::Acatg8C | The DNA fragment containing *Acatg8* with its promoter and terminator was inserted into pEASY-Blunt | This study |
| pEB::CAcatg8 | The cDNA of *Acatg8* was inserted into pEASY-Blunt | This study |
| pEB::Pgpd | The triphosphate dehydrogenase promoter sequence was inserted into pEASY-Blunt | This study |
| pEB::Ter | The terminator of *Acatg8* was inserted into pEASY-Blunt | This study |
| pEB::Gfp | The *GFP* gene was inserted into pEASY-Blunt | This study |
| pEB::GfpN | The *GFP* gene without terminator was inserted into pEASY-Blunt | This study |
| pEB-Rfp | The *RFP* gene was inserted into pEASY-Blunt | This study |
| pEB::PpcbC | The *pcbC* promoter sequence was inserted into pEASY-Blunt | This study |
| pEB::PAcatg8 | The *Acatg8* promoter sequence was inserted into pEASY-Blunt | This study |
| pEB::pcbC | The *pcbC* cDNA sequence was inserted into pEASY-Blunt | This study |
| pEB::PcefD2 | The *cefD2* promoter sequence was inserted into pEASY-Blunt | This study |
| pEB::cefD2 | The *cefD2* cDNA sequence was inserted into pEASY-Blunt | This study |
| pEB::cit | The *Accit* sequence was inserted into pEASY-Blunt | This study |
| pEB::CAcatg8 | The *Acatg8* cDNA sequence was inserted into pEASY-Blunt | This study |
| pEB::Acatg8LR | The flanking sequence of *Acatg8* was inserted into pEASY-Blunt | This study |
| pEB::Acatg8LR-*ble* | *ble* was inserted into pEASY-atg8LR | This study |
| pEB::xyl^P^ | The xyl^P^ fragment was inserted into pEASY-Blunt | This study |
| pEB::T | The terminator fragment was inserted into pEASY-Blunt | This study |
| pEB::CAcatg8 | The cDNA of *Acatg8* was inserted into the *Xba*I site of pEASY-Blunt | This study |
| pEGFP-N | plasmid containing the *GFP* gene | Clontech |
| pDsRed-2 | plasmid containing the *RFP* gene | Clontech |
| pJL43-RNAi | plasmid containing the bleomycin resistant gene (*ble*) | [2] |
| pCMV3×Flag-10 | The vector used for subcloning | Sigma |
| pCMV::xyl^P^ | The xyl^P^ fragment was inserted into pCMV | This study |
| pCMV::xyl^P^-T | The terminator fragment was inserted into pCMV-xyl^P^ | This study |
| pCMV::xyl^P^-GFP-T | The *GFP* gene was inserted into pCMV-xyl^P^ | This study |
| pCMV::xyl^P^-GFP-Acatg8-T | *Acatg8* was inserted into pCMV-xyl^P^-GFP-T | This study |
| pCMV::PpcbC-GFP-T | The *pcbC* promoter was inserted into pCMV-GFP-T | This study |
| pCMV::PcefD2-GFP-T | The *cefD2* promoter was inserted into pCMV-GFP-T | This study |
| pCMV::PpcbC-pcbC-GFP-T | The *pcbC* fragment was inserted into pCMV-PpcbC-GFP-T | This study |
| pCMV::PcefD2-GFP-cefD2-T | The *cefD2* fragment was inserted into pCMV-PcefD2-GFP-T | This study |
| pCMV::Pgpd-cit-GFP-T | The *Accit* fragment was inserted into pCMV-Pgpd-GFP-T | This study |
| pCMV::xyl^P^-PAcatg8-GFP-Acatg8-T | The xyl^P^ promoter was inserted into pCMV- PAcatg8-GFP-Acatg8-T | This study |
| pYES2 | The vector used for transformation | Invitrogen |
| pYES2::CAcatg8 | *Acatg8* was inserted into pYES2 | This study |
| pAg1-H3 | The vector used for ATMT | [1] |
| pAgHB | *ble* was inserted into pAg1-H3 | This study |
| pAgB | *Hph* was deleted from pAgHB | This study |
| pAg::Acatg8LR-B | The *Acatg8* DNA fragment containing the upstream and downstream flanking sequence and the bleomycin resistant gene (*ble*) was inserted into pAg1H3 | This study |
| PAg::Acatg8C | The DNA fragment containing *Acatg8* with its promoter and terminator was inserted into pAg1H3  promoter and terminator was inserted into pAg1H3 | This study |
| pAgB::PAcatg8-GFP-Acatg8-T | PAcatg8-GFP-Acatg8-T was inserted into pAgB | This study |
| pAg1H3::PAcAtg8 | The promoter of *Acatg8* was inserted into pAg1-H3 | This study |
| pAg::PAcatg8-GFP-Acatg8-T | PAcatg8-GFP-Acatg8-T was inserted into pAg1-H3 | This study |
| pAg::PcefD2-GFP-cefD2-T | The PcefD2-GFP-cefD2-T fragment was inserted into pAgHB | This study |
| pAg::PpcbC-pcbC-GFP-T | The PpcbC-pcbC-GFP-T was inserted into pAgHB | This study |
| pAg::xyl^P^-GFP-Acatg8-T | The DNA fragment containing xyl^P^-PAcatg8-GFP-Acatg8-T was inserted into pAg1-H3 | This study |

^a^ CGMCC, China General Microbiological Culture Collection Center.

**Table S2** Primers used in this study.

| Primers | Sequence (5’-3’)^a^ |
| --- | --- |
| GFP-F | AGATCTGATGGTGAGCAAGGGCGAGGAGC |
| GFP-R | AGATCTATTTACTTGTACAGCTCGTCCATGC |
| Pgpd-F | AAGCTTTTTAAATATAGGGCGAATTGGAGCTC |
| Ter-R | GGATCCATTTAAATCTGGCTTCGTACATAGAGGCTC |
| Acatg8DF | ATTTAAATTACCCGCCCAACCTCTA |
| Acatg8DR | ATTTAAATCGGACCACCAAACGAAT |
| Acatg8-outF | TTACAAATGCCATAGAACC |
| Acatg8-outR | CAAGTCCGCAGAAAGAA |
| SAcatg8-F | CGAGGGAATGAATGGTGAA |
| SAcatg8-R | CTGGGAGATGGATGATGGAG |
| Ter -F | GGATCCCTGGCTTCGTACATAGAGGCTC |
| Ter -R | GGATCCTCGATGGTGTTTCCGAGTG |
| Atg8RT-F | CGTCATCTGCGAGAAAGTC |
| Atg8RT-R | CCCGTCCTCATCCTTGTG |
| Cit -F | CGATGGCCTCCGTCACCCGCG |
| Cit -R | CGGAATTCGCCAGCTTCTTGACGAGCTCAGC |
| Pro-F | CCCAAGCTTTCCTTCTCGGCGAGGGACGC |
| Pro-R | CCCAAGCTTGATGGCGGTTGGGTAGCTGCAC |
| Acatg8C-F | TTAATTAATCCTTCTCGGCGAGGGACGC |
| Acatg8C-R | TTAATTAATCGATGGTGTTTCCGAGTG |
| Ppcbc-F | AAGCTTGACTGGCAGTCCACTACTGACTG |
| Ppcbc-R | AAGCTTAGGTGACGGTTTGTCCTGCC |
| pcbC-F | GAATTCAATGGGTTCCGTTCCAGTTCCA |
| pcbC-R | GAATTCGCGGTCTGACCATTCTTGTTGATCA |
| PcefD2-F | GAATTCCGAGGTGGGATTAACCGCG |
| PcefD2-R | GAATTCTGGGGATTGCCGTTATGCT |
| cefD2-F | TCTAGAATGGACCCCTCTCGCCCAC |
| cefD2-R | TCTAGACTACAACTTTTCCCGCGAACA |
| EcefD1-F | TAAGCACTTCTGCCCGCG |
| EcefD1-R | GCAAGAGGATTTCGGGAGGT |
| EcefD2-F | TGTACACCCACCTTGCACG |
| EcefD2-R | CATCGTCTCCATCCTCCC |
| EcefEFG1-F | GGAGTAGCGTGGAGGAAAG |
| EcefEFG1-R | CCATCGGTTAAGAAGGTC |
| EcefEFG2-F | CCATCGGTTAAGAAGGTC |
| EcefEFG2-R | CCGACGGCAGCATCTATC |
| xyl^P^-F | AAGCTTATTTAAATGAGGCCGGACAAATTCAGC |
| xyl^P^-R | AAGCTTGGTGTCAGGGTGTTGAAGATGG |
| gfpF | AGATCTGATGGTGAGCAAGGGCGAGGAGC |
| gfpNR | AGATCTTACTTGTACAGCTCGTCCATGC |
| Acatg8F | TCTAGAATGCGCAGCAAGTTCAAG |
| Acatg8R | TCTAGAAGCGCTGCCGAATGT |
| peat-F | TAAACGGATTTAAATTAAATAGGGCGAATTGGAGCTC |
| peat-R | CCTGCAGGTCGACATTAACTGGCTTCGTACATAGAGGCTC |
| RTbrlA-F | CGGAGGGTGGTCACAAAACT |
| RTbrlA-R | CGAACCCACCATCCACCTT |
| RTwetA-F | GACGGCAACAACTGGTGGG |
| RTwetA-R | GGAGTGCGAGGCTGGGTTA |
| RTabaA-F | GCCGAACTTCGTTGCTTGC |
| RTabaA-R | TGACATGGTGGGCTGGATTAG |
| Gapdh-real-F | TACGCAGAACGAAACAACTAACAA |
| Gapdh-real-R | GGACACGACGGGAGTAGCC |
| RTpcbAB-F | CAATACGGTGGGTGTTAGCG |
| RTpcbAB-R | ACCAGTCCGACGTGCAGAAT |
| RTpcbC-F | TCGGTGATATGGGCCATGTAG |
| RTpcbC-R | GTATCGGGGATCTTGTTGGG |
| RTcefD1-F | TGCTGCTCCTGCCCTCAT |
| RTcefD1-R | CGAAGCCGCTCACCAACT |
| RTcefD2-F | AGGAACAAGTCGTCCATCTGC |
| RTcefD2-R | CTTGAGAAGGACCTCTGTGGG |
| RTcefEF-F | CCGTAACCACCAAGGGTATCT |
| RTcefEF-R | CTCCTCGCTTCCGTTCTTGA |
| RTcefG-F | AAGAGCAAACCTGCGATGGA |
| RTcefG-R | TCTGTGCCGTTGATTTCCTTCT |
| RTactin-F | GCTCGGCCAGAATCTTCA |
| RTactin-R | GGCTCCCATCAACCCAAA |
| RT*Sc-*actin-F | CTCGTGCTGTCTTCCCATCTATC |
| RT*Sc-*actin-R | TCCATATCGTCCCAGTTGGTG |

^a^ The underlined nucleotide sequences indicate restriction enzyme site


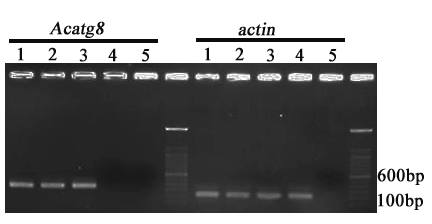


**Fig. S1** Verification of the heterologous complemented strains of Δatg8 by RT-PCR. The plasmid pYES2::*Acatg8* was introduced into the *S. cerevisiae ATG8* mutant (Δatg8) to generate the complemented strains YC1, YC2 and YC3. 1-3, the complemented strains YC1, YC2 and YC3, respectively; 4, ΔATG8; 5, the negative control with water as template. *Acatg8*, DNA fragment was amplified by PCR with primers SAcatg8-F/R; *actin*, DNA fragment was amplified by PCR with primers RTSc-actin-F/R.

**
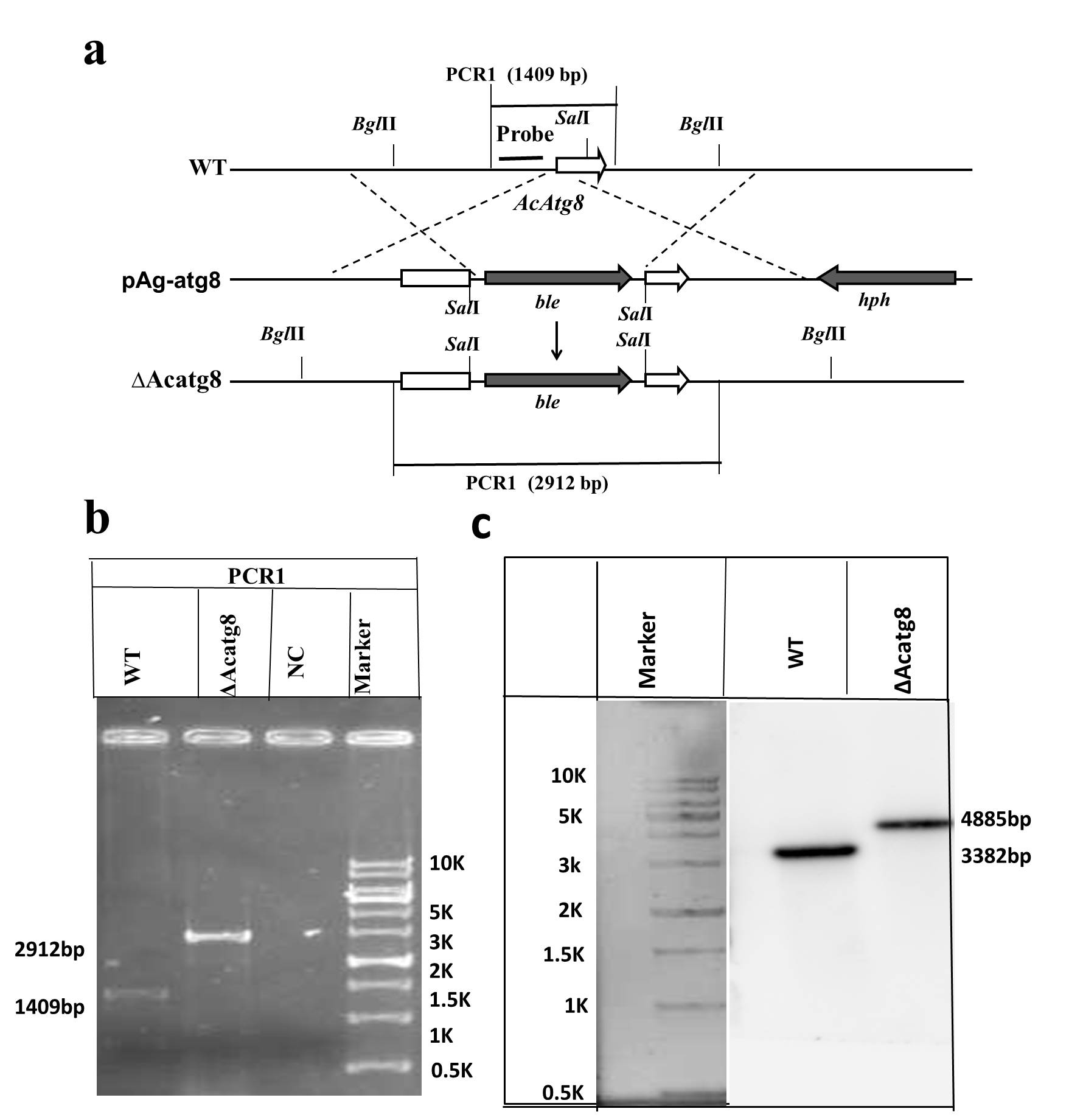
**

**Fig. S2** Construction of the *Acatg8* disruption mutant. (a) Strategy used for construction of the *Acatg8* disruption mutant (ΔAcatg8) via homologous recombination. *hph*, hygromycin phosphotransferase gene; *ble*, bleomycin resistance gene; kb, kilobase pairs; bp, base pairs. (b) Identification of ΔAcatg8 by PCR with primers AcAtg8DF/AcAtg8DR. WT, the *A. chrysogenum* wild-type strain; ΔAcatg8, the *Acatg8* disruption mutant; NC, the negative control; Marker, 1 kb ladder. **(**c) Confirmation of ΔAcatg8 by Southern hybridization. The fungal genomic DNA was digested with *Bgl*II. Probe is indicated by a bar.

**
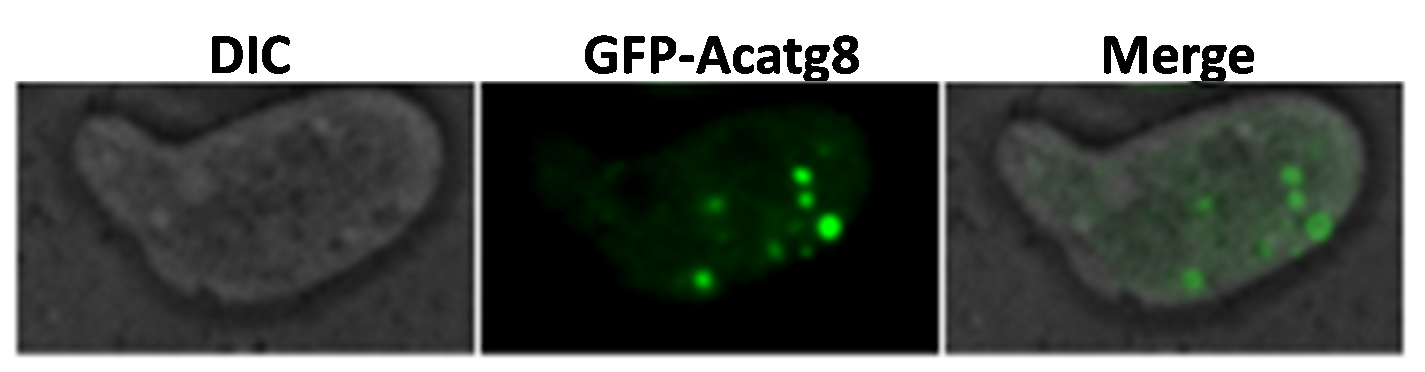
**

**Fig. S3** Localization of AcAtg8 during conidial germination of *A. chrysogenum*. Spores of the *A. chrysogenum* transformant containing pAgB::GFP-Acatg8 were incubated on LPE plates for 4 hours. AcAtg8 fused with GFP was observed during conidial germination. DIC, differential interference contrast; GFP, green fluorescent protein.


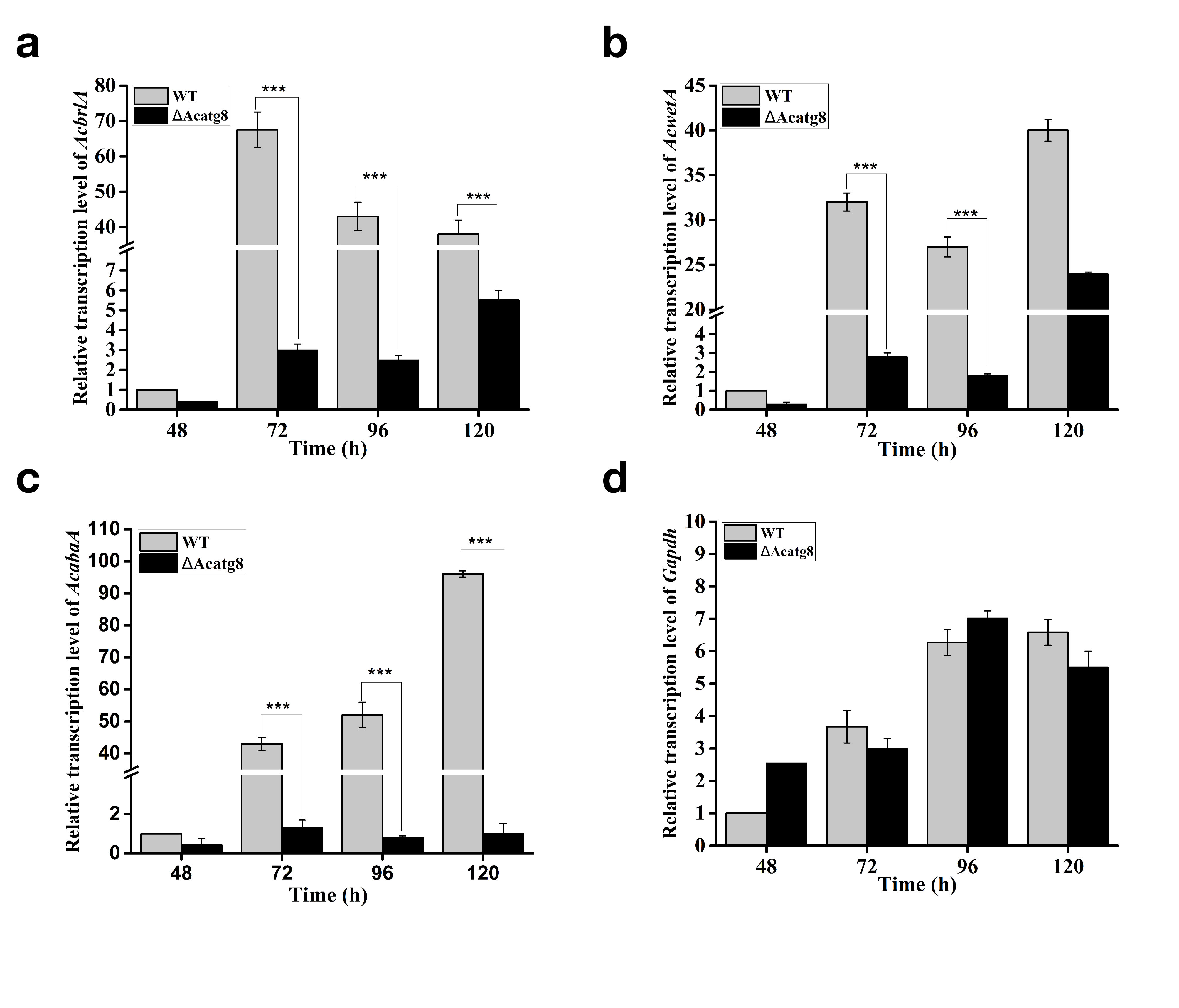


**Fig. S4** Relative transcriptional level of *AcbrlA, AcwetA* and *AcabaA* for conidiation in WT, ∆Acatg8 and Acatg8C. The fungal strains were grown in LPE medium for 120 hours and the mycelia were collected every 24 hours for RNA isolation. Real-time RT-PCR was performed for detecting the transcriptional level of *AcbrlA* (a)*, AcwetA* (b)*,* *AcabaA* (c) and *AcGapdh* (d). The transcript level of *AcGapdh* was used as control. The relative abundance of mRNAs was standardized against to the transcription level of *actin* gene in WT. Error bars represent standard deviations from three independent experiments. The asterix indicates that the differences between strains are significant. p<0.001***; p<0.01**; p<0.05*.


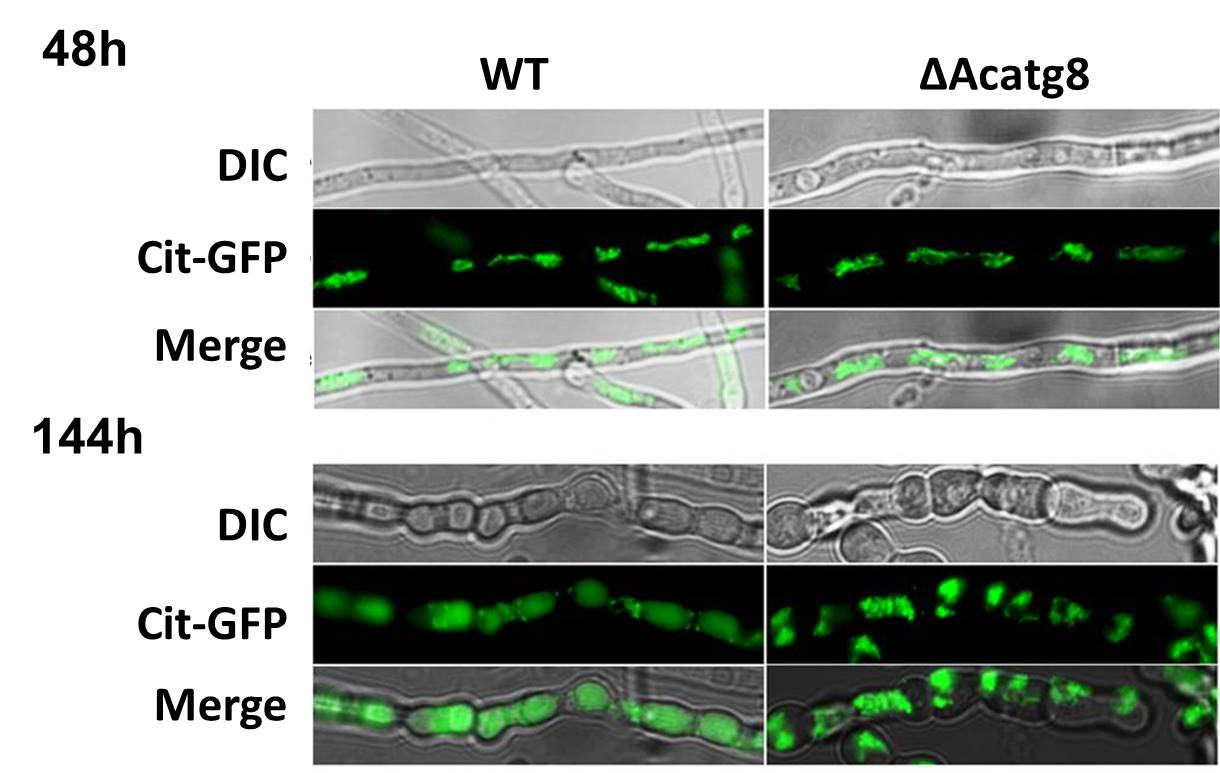


**Fig. S5** Degradation of mitochondria in WT and ΔAcatg8. The degradation of mitochondria was detected by observation of the citrate dehydrogenase fused with GFP. At the early stage (48 h) of fermentation, mitochondrial degradation of WT and ∆Acatg8 did not occur. At the late stage (144 h) of fermentation, vacuoles of WT were filled with fluorescence, indicating a large number of mitochondria were degraded. While there was no fluorescence in the vacuoles of ΔAcatg8, indicating that mitochondria were not degraded in ΔAcatg8.


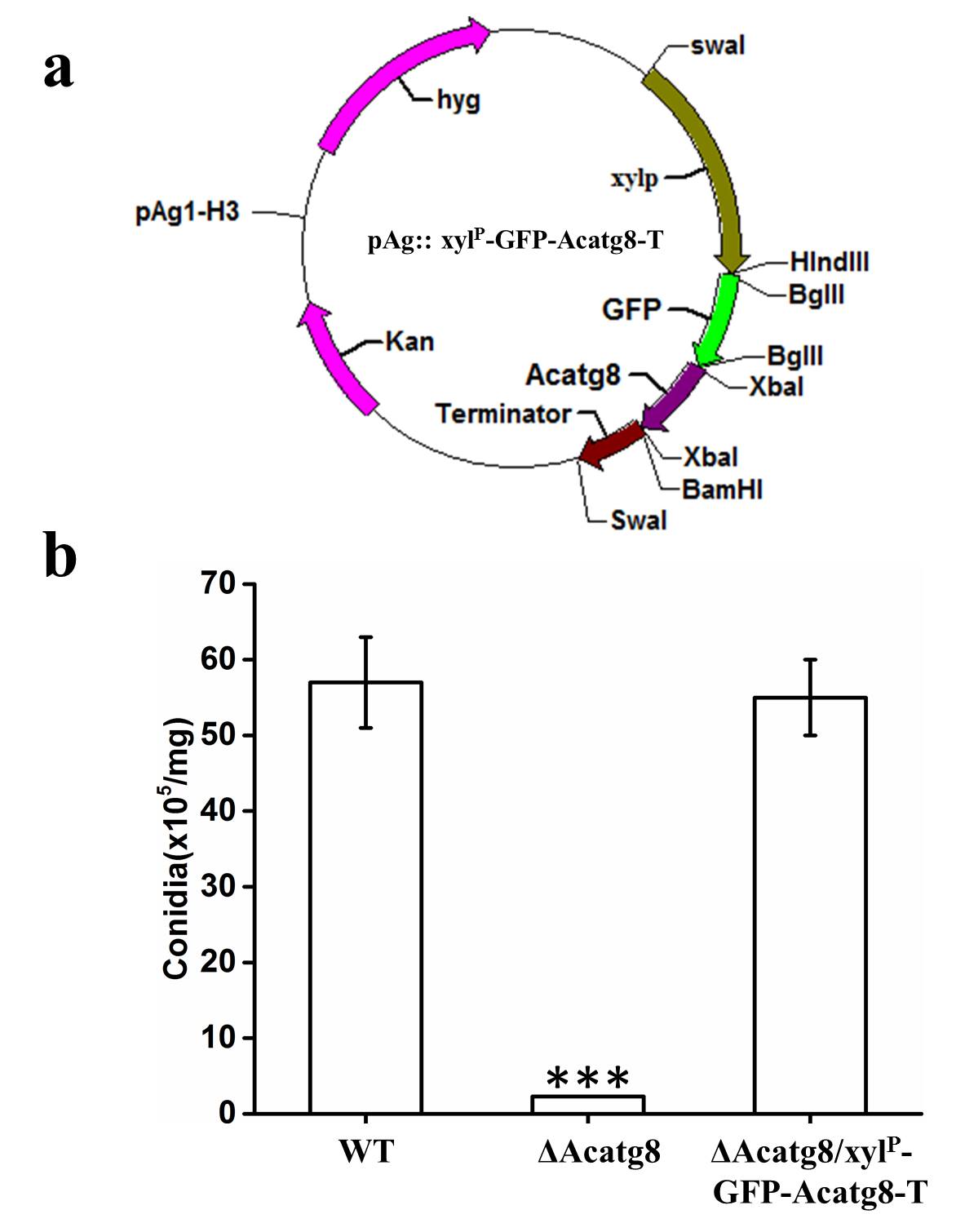


**Fig. S6** Complementation of ∆Acatg8 with *Acatg8* under control of xyl^P^. (a) Construction of the plasmid containing a copy of *Acatg8* under control of the endogenous xylose/xylan-inducible xyl^P^ promoter. (b) Conidial formation in WT, ∆Acatg8 and ΔAcatg8/xyl^P^-GFP-Acatg8-T which were cultured on LPE medium supplemented with 1% xylose for 7 days. Error bars represent standard deviations from three independent experiments. The asterix indicates that the differences between strains are significant. p<0.001 ***; p<0.01 **; p<0.05 *.

**
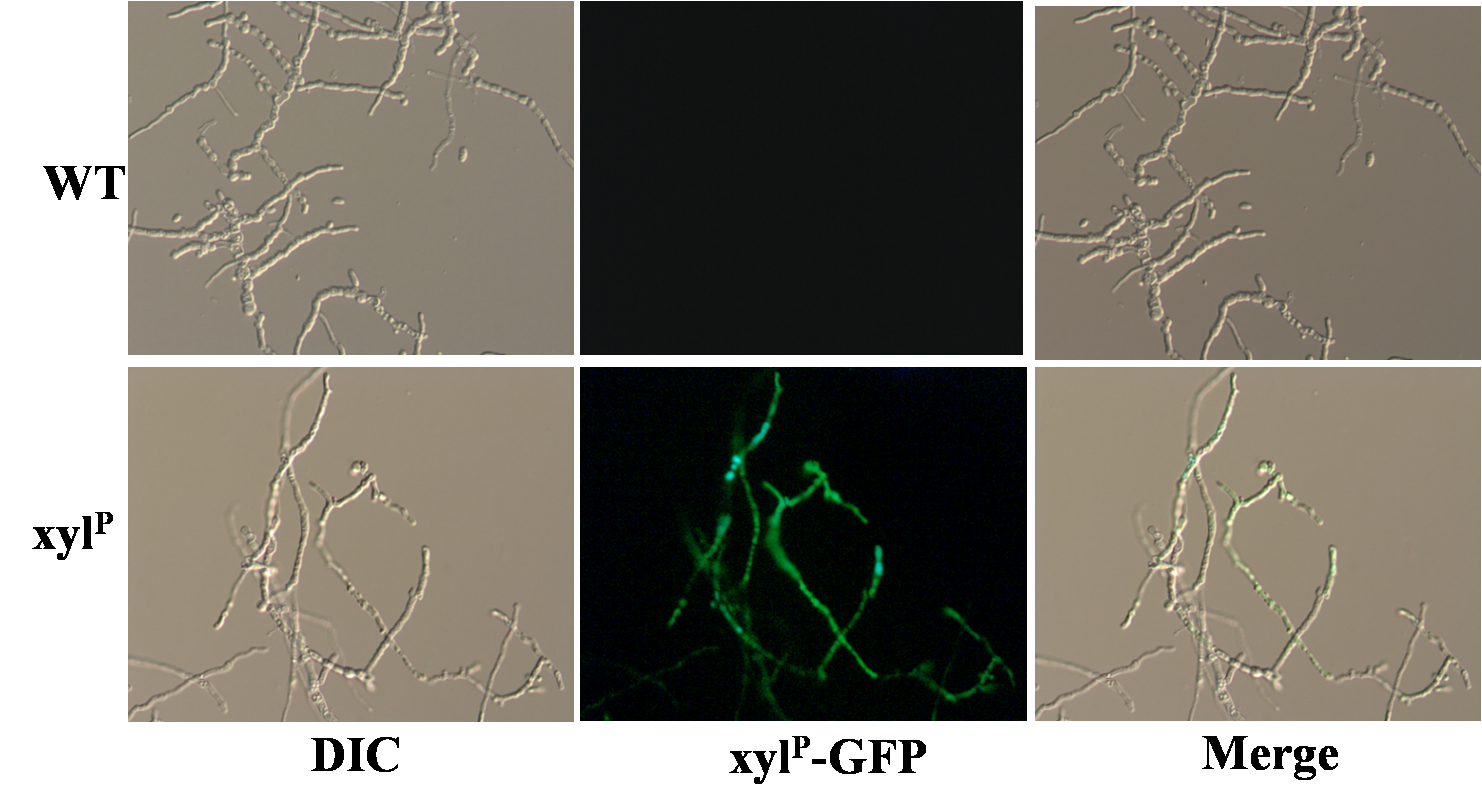
**

**Fig. S7** Inducible expression of *Acatg8* under control of the xyl^P^ promoter. WT, the wild-type strain; xyl^P^, ΔAcatg8/pAg::xyl^P^-GFP-Acatg8-T. The fungal strains were cultured for 6 days in the modified MDFA medium supplemented with 1% xylose. Fluorescence was observed under microscope. DIC, differential interference contrast; xyl^P^-GFP, green fluorescent protein.


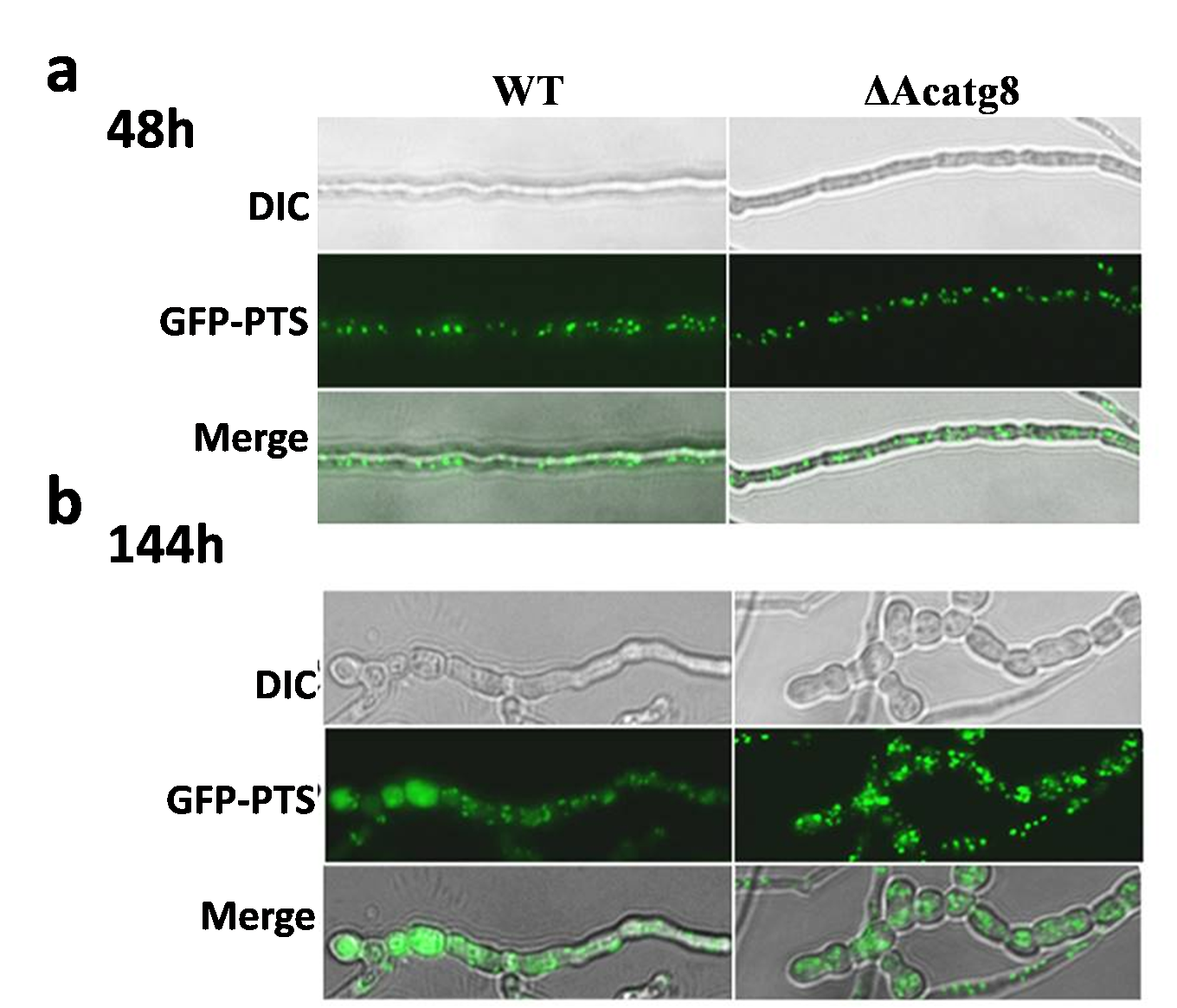


**Fig. S8** Degradation of peroxisomes in WT and ΔAcatg8 during fermentation. Peroxisomes were labeled by a green fluorescent protein (GFP) with a peroxisomal targeting signal (PTS) at its C-terminus. The numbers of peroxisomes in WT and ΔAcatg8 were almost the same at the early stage (48 h) of fermentation and no degradation was observed. At the late stage (144 h) of fermentation, the vacuoles of WT were filled with fluorescence, indicating a large amount of peroxisomes were degraded. Whereas, there was no fluorescence in the vacuoles of ΔAcatg8, and more peroxisomes were accumulated in the cytoplasm.


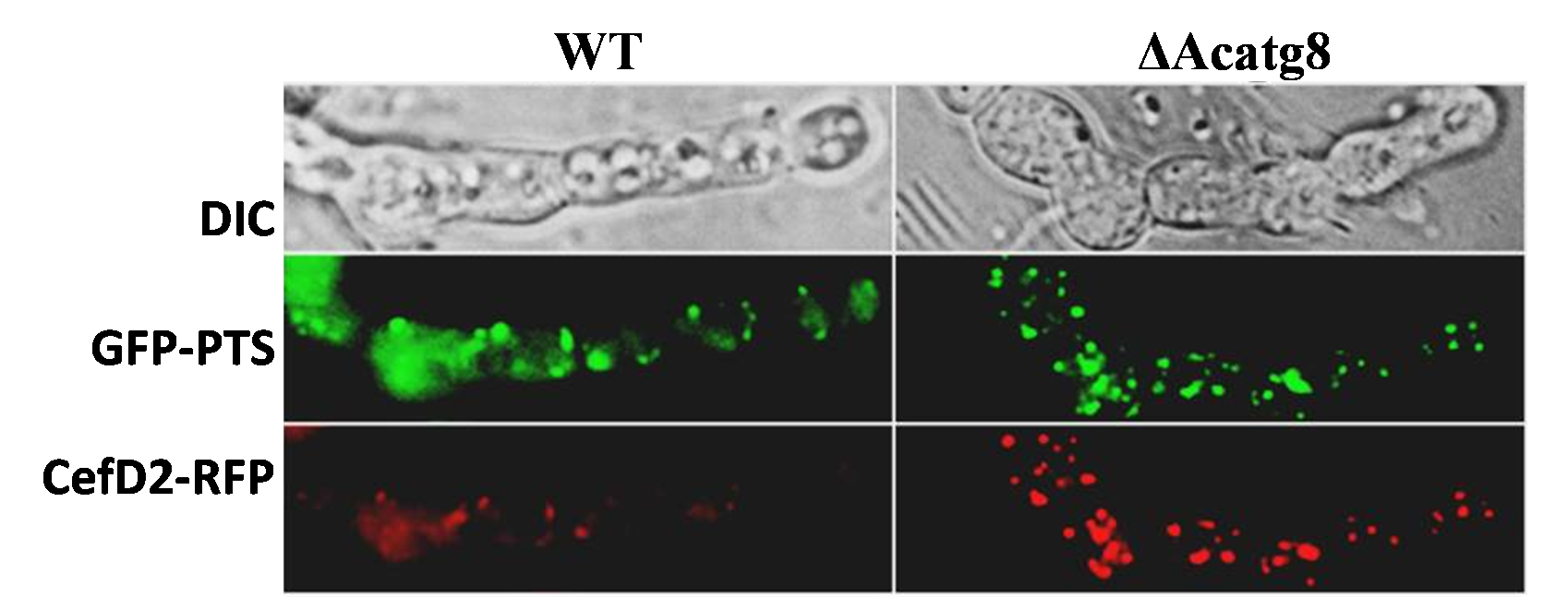


**Fig. S9** Degradation of CefD2 in WT and ΔAcatg8 during fermentation. The peroxisomes were labeled by a green fluorescent protein (GFP) with a peroxisomal targeting signal (PTS) at its C-terminus and CefD2 was labeled by a red fluorescent protein (RFP). Fluorescence observation demonstrated that CefD2 was localized to peroxisomes. After 144 hours fermentation, the peroxisomes and the CefD2 protein were degraded extensively in WT, while a large number of peroxisomes and CefD2 were accumulated in ΔAcatg8.

**REFERENCES**

[1] Khang CH, Park SY, Rho HS, Lee YH, Kang S. Filamentous fungi (*Magnaporthe grisea* and *Fusarium oxysporum*). Methods Mol Biol. 2006; 344: 403-420.

[2] Ullan RV, Godio RP, Teijeira F, Vaca I, Garcia-Estrada C, Feltrer R, et al. RNA-silencing in *Penicillium chrysogenum* and *Acremonium chrysogenum*: validation studies using beta-lactam genes expression. J Microbiol Methods. 2008; 75**:**209-218.
